# Toxicogenomic and Phenotypic Analyses of Bisphenol-A Early-Life Exposure Toxicity in Zebrafish

**DOI:** 10.1371/journal.pone.0028273

**Published:** 2011-12-14

**Authors:** Siew Hong Lam, Mya Myintzu Hlaing, Xiaoyan Zhang, Chuan Yan, Zhenghua Duan, Lin Zhu, Choong Yong Ung, Sinnakaruppan Mathavan, Choon Nam Ong, Zhiyuan Gong

**Affiliations:** 1 Department of Biological Sciences, National University of Singapore, Singapore, Singapore; 2 NUS Environmental Research Institute (NERI), National University of Singapore, Singapore, Singapore; 3 College of Environmental Science and Engineering, Nankai University, Tianjin, China; 4 Genome Institute of Singapore, Agency for Science Technology and Research, Singapore, Singapore; 5 Department of Epidemiology and Public Health, National University of Singapore, Singapore, Singapore; University of Birmingham, United Kingdom

## Abstract

Bisphenol-A is an important environmental contaminant due to the increased early-life exposure that may pose significant health-risks to various organisms including humans. This study aimed to use zebrafish as a toxicogenomic model to capture transcriptomic and phenotypic changes for inference of signaling pathways, biological processes, physiological systems and identify potential biomarker genes that are affected by early-life exposure to bisphenol-A. Phenotypic analysis using wild-type zebrafish larvae revealed BPA early-life exposure toxicity caused cardiac edema, cranio-facial abnormality, failure of swimbladder inflation and poor tactile response. Fluorescent imaging analysis using three transgenic lines revealed suppressed neuron branching from the spinal cord, abnormal development of neuromast cells, and suppressed vascularization in the abdominal region. Using knowledge-based data mining algorithms, transcriptome analysis suggests that several signaling pathways involving ephrin receptor, clathrin-mediated endocytosis, synaptic long-term potentiation, axonal guidance, vascular endothelial growth factor, integrin and tight junction were deregulated. Physiological systems with related disorders associated with the nervous, cardiovascular, skeletal-muscular, blood and reproductive systems were implicated, hence corroborated with the phenotypic analysis. Further analysis identified a common set of BPA-targeted genes and revealed a plausible mechanism involving disruption of endocrine-regulated genes and processes in known susceptible tissue-organs. The expression of 28 genes were validated in a separate experiment using quantitative real-time PCR and 6 genes, *ncl1, apoeb, mdm1, mycl1b, sp4, U1SNRNPBP* homolog, were found to be sensitive and robust biomarkers for BPA early-life exposure toxicity. The susceptibility of *sp4* to BPA perturbation suggests its role in altering brain development, function and subsequently behavior observed in laboratory animals exposed to BPA during early life, which is a health-risk concern of early life exposure in humans. The present study further established zebrafish as a model for toxicogenomic inference of early-life chemical exposure toxicity.

## Introduction

Bisphenol-A (BPA) used in the manufacture of polycarbonate plastic and epoxy resin is among the highest-production-volume chemicals in the world [1]. It is an important contaminant due to its ubiquitous presence and the increased exposure of humans and organisms to BPA via environment and food chain. The concern on the ecological impact of BPA has been increasingly raised in the past few years as more studies reveal ontogenetic and endocrine disruptions by BPA on aquatic organisms at environmental relevant concentrations [2], [3]. In line with this concern are the controversies due to conflicting reports and equivocal findings regarding potential human health effects of early-life exposure to BPA [4]–[6] at the level similar to those used in rodent studies, in which neuro-developmental effects, defect in reproductive tissue development, and predisposition to preneoplastic lesions of the mammary gland and prostate gland in adult life have been reported [7]–[11]. Although there had been many BPA studies involving rodents including several well-designed multi-generation studies [6], [7], there is still limited *in vivo* toxicogenomic information on BPA-induced toxicity during early life. This may be partly due to the protracted *in utero* development of rodents which restrict accessibility and experimental manipulation.

The zebrafish is a premier model for both developmental [12] and toxicological [13] studies. It is also increasingly used for modeling human diseases [14] and for pre-clinical drug screening [15]. The availability of zebrafish in large numbers, its small size and easy husbandry makes the zebrafish a more cost-effective model than the rodent models for toxicological studies. This is important in view that there is a great demand for cost-effective and ethically acceptable approaches to evaluate the toxicity of pharmaceuticals, industrial chemicals and effluents by regulators and industry, and there are strong advocates for using zebrafish to reduce the use of rodent models for such purposes [16], [17]. Presently, zebrafish embryo is included as one of the principal test organism within the Organization for Economic Cooperation and Development test guidelines for endocrine-disruptive chemicals [18]. BPA is an endocrine disruptor and the aquatic environment is a major sink of BPA due to leaching from plastic debris, landfill wastes and sewage effluent [2], [3], thus making zebrafish a relevant model for investigating BPA toxicity.

Owing to the conserved developmental program within vertebrates, fish and mammals share many similar developmental processes. Many of the genes or molecules with essential functions found in humans such as those involved in developmental processes and toxicological responses are also found in zebrafish. We and others have shown that zebrafish is responsive to chemicals, such as small molecules, drugs and environmental toxicants, in a similar manner as mammals [19]–[23]. Consequently, zebrafish is also susceptible to chemical-induced pathology similar to those observed in mammals [22], [24]. The availability of many zebrafish transgenic and enhancer trap lines with fluorescent reporter genes expressed in specific tissues/organs could help in visualizing and real-time monitoring of chemical-induced toxicity changes in live animals [25]. In addition, the amenability of the zebrafish system to various molecular techniques and the vast genomic resources, including the near-completed zebrafish genome project and availability of zebrafish microarrays, makes zebrafish a highly versatile system for toxicogenomic studies [26], [27].

Previously, we have demonstrated that transcriptomic data generated from whole-adult zebrafish, despite loss of weak signals and loss of signal response location, is able to capture strong and well-represented expression signals that are sufficiently robust for predictive chemical biology, for discovering biomarkers and major signaling pathways, as well as useful for human health-risk and biological insight inference [19]. In this study, we applied a similar approach on whole zebrafish larvae to capture strong and well-represented expression signals affected by early-life exposure to BPA. We performed toxicogenomic analysis with the aim of inferring signaling pathways, biological processes, physiological systems and potential biomarkers that may be affected by early-life exposure to BPA in zebrafish. We corroborated the toxicogenomic inferences with phenotypic end-points in wild-type and specific transgenic lines. We further identified a common set of BPA affected genes and performed a focused knowledge-based connectivity network analysis to derive plausible mechanistic insights of BPA early-life exposure toxicity. Finally, we carried out an independent validation experiment by treating new batches of developing zebrafish and validated 28 genes via real-time quantitative PCR whereby 6 were found to be sensitive and robust, hence potentially good biomarkers for BPA early-life exposure toxicity. We found *sp4* which encodes a transcription factor to be susceptible to BPA perturbation and may account for the altered brain development, function and subsequently behavior observed in laboratory animals exposed to BPA during early life. This study demonstrated the potential of using zebrafish as a model for toxicogenomic inference of early-life exposure toxicity through BPA-induced cumulative phenotype and transcriptome analyses.

## Results and Discussion

### Phenotypic analysis of BPA early-life exposure toxicity in wild-type zebrafish

Developing zebrafish were exposed to nominal concentration of 50, 100, 500, 1500 and 4500 µg/L BPA (0.22 µM to 19.71 µM) with final concentration 0.05% (v/v) ethanol (vehicle) in egg water for 7 days from 3 hours post-fertilization (hpf) onwards. Control fish were maintained in egg water with vehicle alone and media was renewed daily. The chosen BPA concentrations spanned from environmental-relevant high concentrations (>1000 µg/L) found in landfill leachate and sewage treatment effluent sources [2], [3] to the range of a recent proposed total allowable concentration (100 µg/L) for drinking water [1]. This embryo-larval 7-day static renewal test, adopted from short term methods for estimating chronic toxicity in freshwater organisms [28], allows for assessment of the cumulative adverse teratogenic effects of chemical(s) on the development and physiology of the organism during early life.

**Figure 1:**
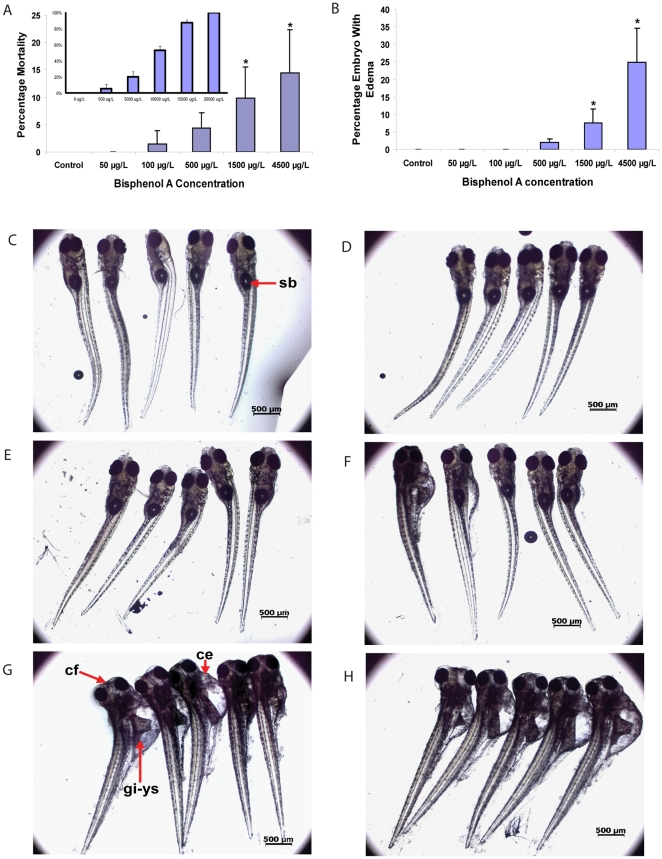
Phenotype analysis of BPA early-life exposure toxicity in wild-type zebrafish larvae. (A) Percentage of mortality and (B) percentage of larvae with edema when developing zebrafish were exposed to BPA at 50, 100, 500, 1500 and 4500 µg/L BPA or vehicle control for 7 days from 3 hour post-fertilization (hpf) onwards. Error bars represent mean ± SD. Asterisk indicate significant (*P*<0.05) differences when compared to control group (*n* = 4 replicate batches of embryos; each treatment group consisted of 50 embryos). Inset in (A) represents another set of acute toxicity experiment performed under similar conditions indicating percentage of mortality for developing zebrafish exposed to BPA at 500, 5000,10000, 15000 and 20000 µg/L. Representative samples of zebrafish larvae exposed to: (C) vehicle (control), (D) 50 µg/L, (E) 100, (F) 500, (G) 1500, and (H) 4500 µg/L, of BPA for 7 days from 3 hpf. Adverse toxic effects was observed at higher frequency in 1500 and 4500 µg/L of BPA which include cardiac edema (ce), cranio-facial abnormality (cf) and swimbladder (sb) development/inflation problem. Scale bar = 500 µm.

In a total of four experimental replicates, statistically significant mortality (**Figure 1A**) and cardiac edema (**Figure 1B**) were observed in developing zebrafish larvae treated with 1500 and 4500 µg/L BPA when compared to control (vehicle) group (T-test *P*<0.05; each treatment group consists of 50 embryos and the experiment was repeated four times; *n* = 4). No statistically significant (T-test *P*>0.05) adverse effects were observed in developing zebrafish treated with BPA at 500 µg/L or lower. In addition, fish with cardiac edema also appeared to have cranio-facial abnormality [broad-headed (brachycephalic) and lacks anterior lower jaw protrusion], problems with swimbladder development/inflation and apparent gastro-intestinal abnormalities and partial yolk-sac re-absorption (**Figure 1C–H**), resembling those that we [29] and others [30] have reported previously in thyroid hormone studies using developing zebrafish. Fish with ‘curved-tail-down’ phenotype occurring at higher concentration of BPA suggesting neuro-muscular problem as previously reported [31], [32] were occasionally observed (1–2%) at 1500 and 4500 µg/L BPA (not shown). Fish treated with 1500 and 4500 µg/L BPA appeared to be ‘listless’ (poor tactile response) and some exhibited abnormal swimming behavior that are likely associated with swimbladder and/or neuro-muscular problems.

### Phenotypic analysis of BPA early-life exposure toxicity in transgenic zebrafish

**Figure 2:**
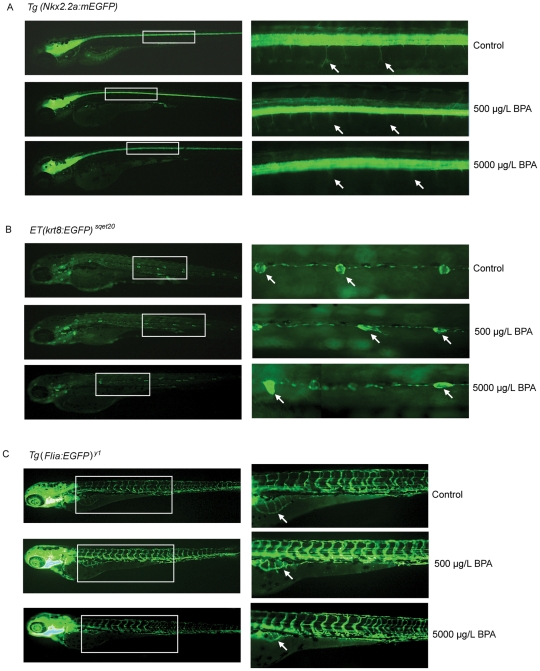
Phenotype analysis of BPA early-life exposure toxicity in GFP transgenic fish larvae. (A) *Tg(Nkx2.2a-mEGFP)* zebrafish larvae exposed to vehicle (control), 500 µg/L BPA and 5000 µg/L BPA. Panel on the right represent the inset region marked by white-outlined box in the corresponding left panel. White arrows indicate axon branching which are normal in control fish but marginally and severely affected in fish exposed to 500 and 5000 µg/L BPA, respectively. (B) *ET(krt8-EGFP)^sqet20^* zebrafish larvae exposed to vehicle (control), 500 µg/L BPA and 5000 µg/L BPA. Panel on the right represent the inset region marked by white-outlined box in the corresponding left panel. White arrows indicate neuromast cells which are rosette circular-like shape in control fish but severely deformed in fish exposed to 500 and 5000 µg/L BPA. (C) *Tg(Flia-EGFP)^y1^* zebrafish larvae exposed to vehicle (control), 500 µg/L and 5000 µg/L BPA. White arrow indicate vascularization in abdominal region which is normal in control fish but suppressed in fish exposed to 500 and (R) 5000 µg/L BPA.

To help better visualize and assess some of the changes in specific tissue-organs as a result of early-life exposure to BPA, we also employed three transgenic lines [*Tg(Nkx2.2a-mEGFP), Tg(Flia-EGFP)^y1^, and ET(krt8-EGFP)^sqet20^*; see **Table S1** for information on the transgenic lines]. However, the transgenic lines appeared to be more sensitive to BPA toxicity as the fish succumbed to BPA at a higher rate (data not shown) compared to the wild-type fish. Hence after preliminary assay optimization, we exposed the fish to 50, 500 and 5000 µg/L BPA for 5 days. Interestingly, *Tg(Nkx2.2a-mEGFP)* revealed that dorsal and ventral growth and branching of axons from the spinal cord were inhibited in fish treated with 5000 µg/L but mildly suppressed in fish treated with 500 µg/L (**Figure 2A**). Similar inhibition of axon growth and branching has been observed in *Tg(Nkx2.2a-mEGFP)* line treated with 17-beta estradiol but not with mefenamic acid (a non-steroidal anti-inflammatory drug) (**Figure S1**) suggesting specific estrogenic neurotoxicity. The findings further suggest that the development of the central nervous system, especially the later stages involving growth and branching of axons, is sensitive to BPA toxicity and this may be related to BPA estrogenic activity although we could not exclude other mode of neurotoxicity that can induce similar phenotype. The *ET(krt4-EGFP)^sqet20^* showed that neuromast cells along the lateral line which normally appeared in a rosette-like circular shape in control fish were deformed with irregular shape in fish exposed to 500 µg/L and 5000 µg/L BPA (**Figure 2B**) suggesting that the neuro-sensory system is affected in these fish. In addition, *Tg(Flia-EGFP)^y1^* further revealed that vascularization in the abdominal region (swimbladder and of the gastro-intestinal region) were also suppressed in a dose-dependent manner at 500 and 5000 µg/L (**Figure 2C**). The overall findings indicate that BPA caused dose-dependent adverse effects as a result of early-life exposure toxicity in transgenic zebrafish exposed to 500 µg/L and 5000 µg/L but no apparent adverse abnormalities were observed in transgenic fish exposed to 50 µg/L of BPA in all experiments (data not shown). The ability to observe phenotypic abnormalities easily is an advantage of the zebrafish model and these abnormalities would provide corroboration as ‘phenotypic-anchoring’ points [33] for subsequent transcriptome analysis into BPA early-life exposure toxicity.

### Microarray data analysis of BPA early-life exposure toxicity in wild-type zebrafish

To determine the levels of toxicity of BPA used for microarray experiments, zebrafish fry were also exposed to higher concentrations of BPA and we estimated that the LC50 is around 8.6 mg/L under our conditions (**Figure 1A** inset). Based on the dose-dependent phenotypic effect observed in wild-type and transgenic zebrafish, BPA concentrations of 500 µg/L, 1500 µg/L and 4500 µg/L (corresponding to LC5 to LC15) were used for the microarray experiment to obtain toxicogenomic information for inferring signaling pathways, biological processes, physiological systems and potential biomarkers that may be affected by early-life exposure to BPA toxicity in zebrafish. Similar experimental design and conditions were employed; 50 larvae were pooled for one biological replicate and the experiment was repeated several times in order to obtain sufficient materials for 4–5 biological replicates used in the microarray hybridization.

**Figure 3:**
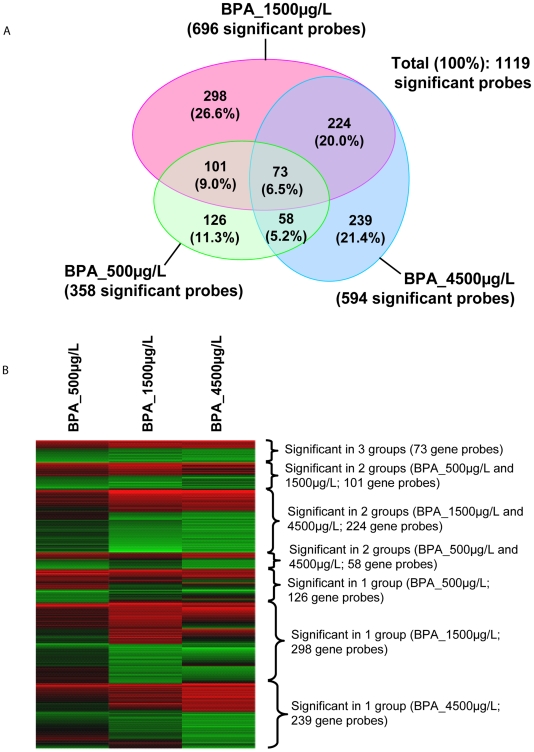
Transcriptomic changes induced by BPA early-life exposure toxicity. (A) Venn-diagrams showing number (percentage) of genes that were considered significantly deregulated among the three treatment groups (BPA_500 µg/L, BPA_1500 µg/L and BPA_4500 µg/L). B) Expression profiles of gene probes categorized based on their significant deregulation in one, two and three treatment groups. The colored cells reflect the mean expression level of four biological replicates in each concentration group when compared to the control (vehicle) group (*n* = 4). Shades of red cells reflect up-regulated, green cells reflect down-regulated and black cells reflect unchanged, expression levels when compared to control (vehicle) group.

Microarray data from a total of 18 arrays, consisting of 5 biological replicate arrays for each control and 500 µg/L groups and 4 biological replicate arrays for each 1500 µg/L and 4500 µg/L groups, were used for subsequent transcriptome analysis. The transcriptome data were subjected to Lowess normalization followed by One-Way ANOVA. After One-Way ANOVA and False Discovery Rate (FDR) adjustment (see Materials and Methods), a total of 1119 genes (with unique GenBank ID) were found to have less than 20% FDR. Subsequent t-test between each treatment group and the control group yielded 358, 696 and 594 genes considered as significantly (ANOVA FDR<20% and T-test *P*<0.05) deregulated within the respective treatment groups of 500, 1500 and 4500 µg/L BPA when compared to control group (**Figure 3A**). The number of deregulated genes increased by two-fold between 500–1500 µg/L suggesting a BPA concentration-dependent response, followed by a slight decrease (∼15%) in the 4500 µg/L group suggesting an onset BPA-induced toxicity affecting gene expression. After accounting for overlapping genes, we found that 73 genes (6.5% of 1119 genes) were significantly deregulated in all three treatment groups while 383 (34.2%) genes were found to be significant in two treatment groups and 663 (59.3%) genes were only significant in one of the treatment group (**Figure 3A**). Even so, it is worth mentioning that majority of these genes demonstrated mean expression profiles that are consistently up- or down-regulated (although may not be statistically significant) or in a concentration-dependent response trend in all the three BPA concentrations when compared to the control group (**Figure 3B**). It is also noteworthy that the levels of *vitellogenin1 (vtg1)* mRNAs was up-regulated 2-fold (p-value = 0.08) at 4500 µg/L BPA and *vitellogenin3* (*vtg3*) mRNAs was significantly (p-value<0.05) up-regulated 2.3-fold and 3.4-fold at 1500 µg/L and 4500 µg/L BPA, respectively, when compared to the control group (data not shown). This confirms that our microarray data were able to capture significant estrogenic effects as indicated by the induction of *vitellogenin* mRNAs. Our data corroborated with the study by Muncke et al [34] which reported up to 50-fold induction of *vtg1* mRNAs by BPA by using a more sensitive real-time PCR assay.

### Independent validation of microarray data and identification of potential biomarkers

**Figure 4:**
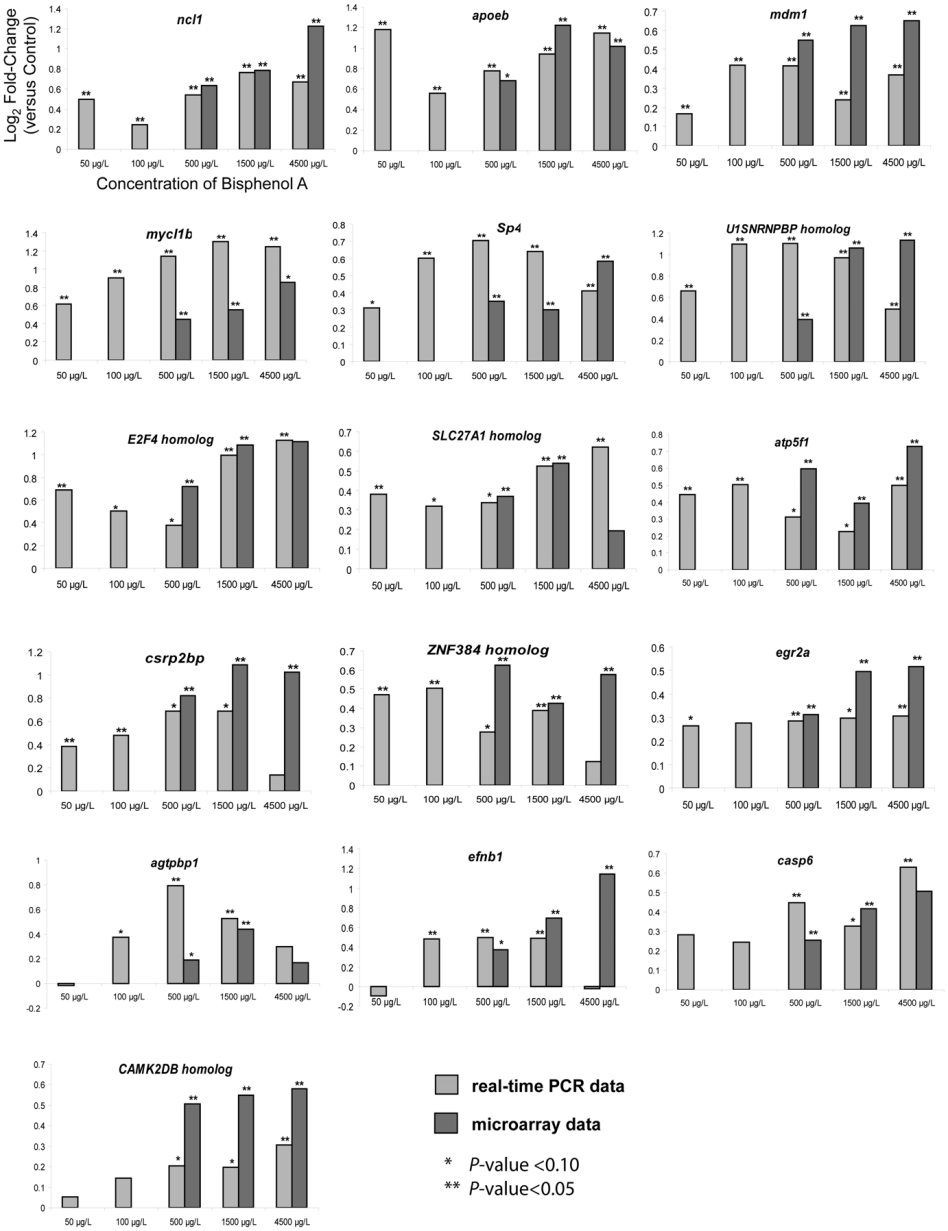
Transcriptomic analysis and inference of BPA early-life exposure toxicity. Selected top functional sub-categories within (A) molecular and cellular function, (B) physiological system development and function, (C) disease and disorder, and (D) canonical pathway, which were all significantly enriched (except for those labeled with asterisk ‘*’where Fisher's Exact Test *P*>0.05) with human homologs of zebrafish genes deregulated in BPA_500 µg/L, BPA_1500 µg/L and BPA_4500 µg/L groups. Refer to Additional Files 2–5 for more information. The figure legend and Y-axis title for the histogram in (A) are applicable to all other histograms.

In order to validate the microarray data and identify potential biomarkers for BPA early-life exposure toxicity, we conducted an independent validation experiment by treating new batches of developing zebrafish similar to the earlier acute toxicity experiment (*n* = 5–6 biological replicates; each biological replicate consists of five pooled larvae). Total RNA was extracted from the pooled larvae of each treatment group and reverse-transcribed to cDNA for real-time PCR validation of 28 genes selected from those that were significant in 2–3 treatment groups. In general, the PCR data showed good concordance with the microarray data in terms of the expression direction (up- or down-regulation) although the fold-change levels of genes compared to their respective controls were not always similar between the PCR and microarray data (**Figure 4** **and** **5**). Nevertheless, they served to validate the expression pattern of these genes captured by the microarray experiment. This further provides greater confidence of the subsequent transcriptome analysis,

**Figure 5:**
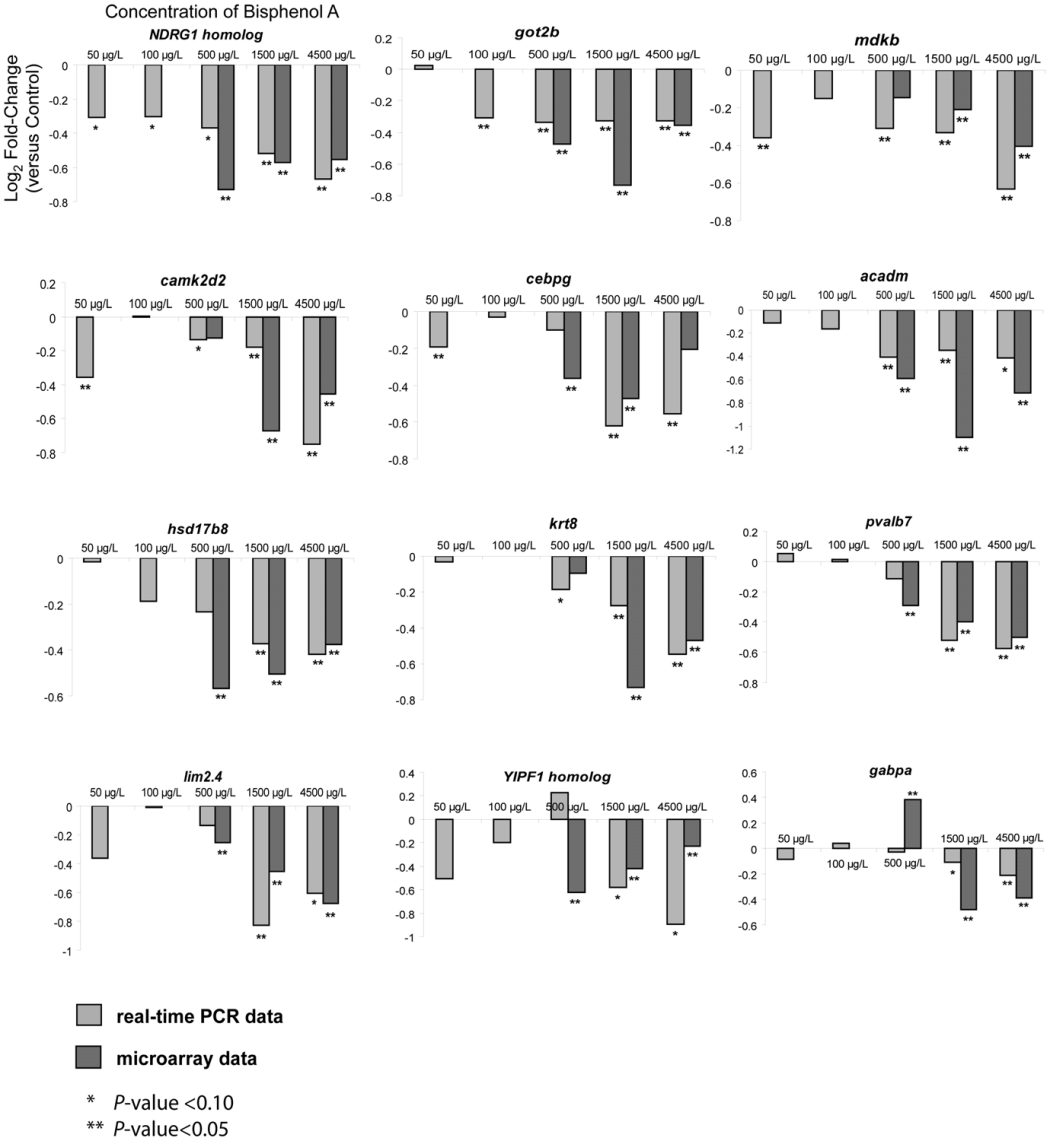
Connectivity network for common-targeted genes with enriched functional subcategories. The network shows the connectivity between significantly (Fisher's Exact Test *P*<0.05) enriched functional subcategories with the endocrine-regulated human homologs of zebrafish genes that were commonly deregulated in all the three treatment groups (BPA_500 µg/L, BPA_1500 µg/L and BPA_4500 µg/L). Refer to Additional Files 6 and 7 for more information.

Among the 28 validated genes (16 up-regulated; 12 down-regulated), 16 were deregulated (T-test *P*<0.10), including 6 (*ncl1, apoeb, mdm1, mycl1b, sp4, U1SNRNPBP* homolog) that were up-regulated and 2 (*got2b, mdkb*) that were down-regulated significantly (T-test *P*<0.05), in at least 4 concentration groups. The remaining 12 genes were deregulated (*P*<0.10) in 2–3 concentration groups. There were also 12 genes that were deregulated (*P*<0.10), including 8 (*ncl1, apoeb, mdm1, mycl1b, U1SNRNPBP* homolog, *atp5f1, csrp2bp, ZNF384* homolog) which were significantly (*P*<0.05) up-regulated, in both 50 and 100 µg/L BPA. Based on these findings, we proposed 6 genes, *ncl1, apoeb, mdm1, mycl1b, sp4, U1SNRNPBP* homolog, which were up-regulated in at least 4 concentration groups (*P*<0.05) as well as in both 50 and 100 µg/L BPA (*P*<0.10) to be sensitive and robust biomarkers for BPA early-life exposure toxicity. It is noteworthy that these 6 genes are deregulated within the proposed total allowable concentration (100 µg/L) of BPA for drinking water [1].

These six genes were further tested under similar experimental regime using 17-beta estradiol and mefenamic acid. We found that all the six genes showed good concordance in terms of statistical significance (p-value<0.1) in the up-regulation trend between samples treated with 17-beta estradiol and BPA at similar concentrations. In fry treated with mefenamic acid at similar concentrations, only three genes (*ncl1, sp4, U1SNRNPBP* homolog) were significantly up-regulated and the other three (*apoeb, mdm1, mycl1b*) were not significant (p-value>0.1) (**Figure S2**). The data suggests that *apoeb, mdm1, mycl1b* may be more specific to up-regulation by 17-beta estradiol and BPA. Since mefenamic acid is not known to have any estrogenic activity and there was no observable phenotype malformation at the concentration used, we could only infer that *ncl1, sp4, U1SNRNPBP* homolog were up-regulated by a different mode of action exerted by mefenamic acid. This, however, do not negate the significance that these 6 genes are indeed sensitive to perturbation by estrogenic compounds during early life of the zebrafish. The expression of *sp4* expression and its role in CNS development and function observed in rodents [35], [36], as well as *SP4* association with various psychiatric disorders in humans [37], [38]. The present study revealed that *sp4* expression is a sensitive target of BPA perturbation, even at relatively low concentration (50 and 100 µg/L BPA) exposure, hence it is susceptible to BPA induced deregulation that could impact brain development, function and consequently behavior as observed in laboratory animals exposed to BPA during early life [7], [8]. This has been a health-risk concern expressed by the latest NTP-CERHR expert panel report [6] with regard to the effects of BPA on the nervous system and behavior as a result of early life exposure in humans. We are currently doing a focused study to characterize BPA-induced perturbation of *sp4* expression in CNS of zebrafish embryos. Likewise, given the known association of the corresponding human homolog of the zebrafish genes such as *apoeb* with lipid metabolism and cardiovascular, neurological, reproductive, immune disorders, *mycl1b* with cancer, *ndgr1* with cancer, reproductive and neurological disorders, hence chronic early-life deregulation of these genes warrant further investigations.

### Transcriptome analysis and inference of BPA early-life exposure toxicity

**Figure 6:**
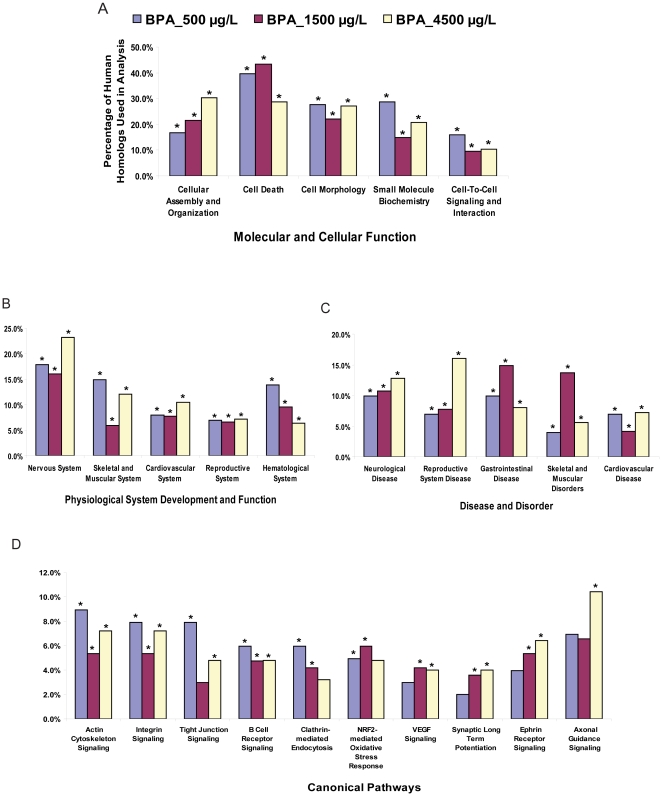
Real-time PCR validation of selected up-regulated zebrafish genes. Using samples from a separate validation experiment (n = 5–6 biological replicates; each replicate consist of five pooled larvae), selected up-regulated genes were validated by real-time PCR and compared with corresponding microarray data. Axis titles on the upper left histogram and figure legends are applicable to all histograms.

Since human databases have better annotations, and most available gene expression analysis software do not support zebrafish data, it is useful to map zebrafish genes to human homologs for knowledge-based data mining. Moreover, such mapping would facilitate comparison of fish data with higher vertebrate data (human/mouse) to capture conservation and to infer the findings from zebrafish to humans. Therefore, we mapped the significantly deregulated gene probes from each of the treatment groups to available corresponding human homologs as previously described [24] (see Materials and Methods). We were able to map the 358, 696 and 594 zebrafish genes to 188, 357 and 291 human homologs, respectively, for the 500, 1500 and 4500 µg/L groups. The mapped human homologs of zebrafish genes were used for knowledge-based data mining via Ingenuity Pathway Analysis™ (IPA) software (see Materials and Methods) to obtain insights into the altered biological functions and signaling pathways associated with the deregulated genes, as well as for inferring affected physiological system and potential health-risks. Based on the Ingenuity database, the algorithm associated 101 (for 500 µg/L), 168 (for 1500 µg/L) and 125 (for 4500 µg/L) of the human homologs with four primary categories: (i) molecular and cellular function, (ii) physiological system development and function, (iii) disease and disorder and (iv) canonical pathway (**Figure 6A–D**). Within each primary category, functional sub-categories which were significantly (Fisher's Exact Test *P*<5.0E-02) enriched with the human homologs of zebrafish genes were listed (**Table S2, S3, S4, S5**).

The analysis suggests that molecular and cellular function associated with cellular assembly and organization, cell death, cell morphology, small molecule biochemistry, and cell-to-cell signaling and interaction, were among those significantly enriched with high percentage (9.5%–44%; Fisher's Exact Test *P*<2.6E-05 to 3.6E-02) of the human homologs in all three treatment groups (**Figure 6A**; complete list in **Table S2**). Physiological system development and function related to nervous system was significantly enriched with the highest percentage (16%–23%; Fisher's Exact Test *P*<3.26E-04 to 3.42E-02) of human homologs in all three treatment groups, followed by skeletal and muscular system, cardiovascular system, reproductive system and hematological system (5%–15%; Fisher's Exact Test *P*<1.45E-03 to 3.24E-02) (**Figure 6B** **, Table S3**). Likewise, neurological disease, reproductive system disease, skeletal muscular disorder, and cardiovascular disease were also significantly enriched with relatively high percentage (4%–16%; Fisher's Exact Test *P*<2.48E-04 to 3.6E-02) of human homologs in all three treatment groups in the ‘disease and disorder’ category (**Figure 6C** **, Table S4**).

In addition, we were able to capture deregulated signaling pathways (**Figure 6D** **, Table S5**) that could be associated with the affected cellular functions, physiological systems and phenotype. Specifically, signaling pathways involved in tight junction, actin cytoskeleton, integrin, clathrin-mediated endocytosis, Nrf2-mediated oxidative stress and ephrin receptor were significantly (Fisher's Exact Test *P*<0.05) deregulated by BPA exposure which are known to be associated with cellular death/survival, differentiation, morphology, movement and intercellular signaling. Pathways associated with tight junction, integrin and cytoskeletal actin signaling can modulate cell morphology and motility [39], [40] while deregulation of ephrin receptor signaling is known to affect axon guidance, cardiovascular, skeletal-muscular and craniofacial development [41], [42] as observed in this study. Deregulation of synaptic long-term potentiation and clathrin-mediated endocytosis which affects synaptic transmission [43], coupled with ephrin receptor and axonal guidance are likely associated with the abnormal neurological phenotypes such as suppressed neuron branching observed in *Tg(Nkx2.2a-mEGFP)*, abnormal neuromast cells in *ET(krt8-EGFP)^sqet20^* and poor tactile response and abnormal swimming behavior in wildtype zebrafish that were exposed to higher levels of BPA. Moreover, deregulation of signaling pathways involving ephrin receptor, clathrin-mediated endocytosis, integrin and tight junction together with VEGF signaling [44], [45] may be linked with the cardiac edema and suppressed vascularization in the abdominal region as observed in wildtype and/or transgenic *Tg(Flia-EGFP)^y1^* exposed to higher levels of BPA. While we could infer association with specific signaling pathways based on deregulated genes and phenotypes observed in the study, it would not be possible to know if a deregulated signaling pathway is a cause or an effect of a phenotype. Future studies for both short and long BPA exposures will be required to link the specific pathways in primary response to secondary responses.

### Common targeted-genes underlying BPA early-life exposure toxicity

To further understand the common underlying molecular targets and mechanisms, we examined the biological functions and associations of the 73 common targeted-genes that were deregulated in all the three treatment groups. This group of common targeted-genes deserves special attention as they were consistently deregulated by BPA from low to high concentration.

We were able to map the 73 genes to 38 human homologs whereby gene ontology annotation indicates that the protein they encode include 11 enzymes, 5 transporters, 4 transcription regulators, 2 signal transducers, 1 transmembrane receptor and 1 structural protein while the remaining 14 have functions that are not well-defined, hence categorized as ‘others’ (**Table 1**). Interestingly, 11 (29%) of the human homologs encoding ACADM, BLNK, CPA2, HSD17B8, NDRG1, RND3, SSR2, SLC34A2, and transcription regulators EGR2, GABPA, SP4 have been reported to be regulated by estrogen, and 3 (8%) of the human homologs encoding LRRC4, SLC34A2 and transcription regulator GABPA have been reported to be regulated by thyroid hormone (see references within **Table S6**). Another human homolog encoding transcription regulator ZNF384/NM4 is known to be regulated by parathyroid hormone. Notably, human homolog encoding HSD17B8 known for its key involvement in androgen and estrogen metabolism was down-regulated hence further amplifying the endocrine disruptive effects on the development of reproductive organs and sexual maturation. The findings revealed that BPA early-life exposure toxicity is partly mediated through the targeting of endocrine-regulated genes which could be captured by zebrafish whole-animal toxicogenomics. While it is known that BPA disrupt estrogen and thyroid hormones signaling [46], this study identified a set of endocrine-regulated genes operating during early life development, previously not known to be disrupted by BPA. Although a number of estrogen-regulated genes were identified, canonical estrogen receptor signaling pathway was not enriched in our analysis. The reason could be estrogen signaling occurs at the upstream protein level while we are likely to have captured some of the downstream effects of estrogen signaling at the transcriptome level. This was evident as many estrogen responsive genes are not part of the canonical estrogen-receptor signaling pathway but rather downstream of it. Moreover, BPA has been shown to act weaker (at least 1000-fold weaker than estradiol) on the classical genomic estrogen receptor signaling pathway compared to its non-genomic estrogenic actions [47]–[49], therefore if there is any deregulation of the canonical estrogen receptor signaling pathway at the transcriptome level, the signals may be too weak and masked by stronger downstream signals. It is also worth highlighting that 39% (15/38) of the common deregulated genes (**Table 1**) including 64% (7/11) of them used in the subsequent network analysis were validated by real-time PCR in an independent experiment (**Figure 4** **and** **5**).

**Table 1:** Human homolog of zebrafish genes (38) that were significantly deregulated in all three BPA exposed groups (500 µg/L, 1500 µg/L and 4500 µg/L).

Gene Symbol [Description]	Mean Fold-Change (Log_2_ Ratio)^a^	Function	Endocrine-regulated^b^
CSRP2BP [CSRP2 binding protein]*	0.98	others	
NCLN [nicalin]*	0.88	enzyme (peptidase)	
U1SNRNPBP [U11/U12 snRNP]*	0.86	others	
ETFA [electron-transfer-flavoprotein, alpha polypeptide]	0.68	transporter	
MRPS9 [mitochondrial ribosomal protein S9]	0.67	others	
MDM1 [Transformed 3T3 cell double minute 1]*	0.61	others	
ATP5F1 [ATP synthase, F0 complex, subunit B1]*	0.57	transporter	
CAMK2D [CaM kinase II delta]*	0.55	enzyme (kinase)	
ZNF384 [zinc finger protein 384; CIZ/Nmp4]*	0.54	transcription regulator	Parathyroid
TRAM2 [translocation associated membrane protein 2]	0.52	transporter	
METAP1 [methionyl aminopeptidase 1]	0.45	enzyme (peptidase)	
EGR2 [early growth response 2; Krox20]*	0.44	transcription regulator	E2
SP4 [specificity protein 4]*	0.41	transcription regulator	E2
GABPA [GA binding protein transcription factor, alpha subunit; NRF2a]*	0.38, −0.43^c^	transcription regulator	Thyroid and E2
AXIN1 [axin 1]	−0.22, 0.20^d^	signal transducer	
FEM1B [fem-1 homolog b]	−0.36	others	
FIP1L1 [FIP1 like 1]	−0.38	others	
RND3 [Rho family GTPase 3]	−0.38	enzyme (GTPase)	E2
MOSPD1 [Motile sperm domain containing 1]	−0.40	others	
MID1IP1 [MID1 interacting protein 1]	−0.42	others	
YIPF1 [Yip1 domain family, member 1]*	−0.43	transporter	
SRPX [sushi-repeat-containing protein, X-linked]	−0.46	others	
LIM2 [lens intrinsic membrane protein 2]*	−0.46	others	
BLNK [B-cell linker]	−0.46	signal transducer	E2
HSD17B8 [hydroxysteroid (17-beta) dehydrogenase 8]*	−0.48	enzyme (metabolic)	E2 and E2 metabolism
LRRC4 [leucine rich repeat containing 4]	−0.52	others	Antithyroid (Propylthiouracil)
ACTG1 [actin, gamma 1]	−0.52	structural protein	
CPA2 [carboxypeptidase A2 (pancreatic)]	−0.56	enzyme (peptidase)	E2
XRN2 [5′-3′ exoribonuclease 2]	−0.57	enzyme (nuclease)	
PCID2 [PCI domain containing 2]	−0.58	others	
SLC34A2 [solute carrier family 34, member 2; NAPI2]	−0.59	transporter	E2 and Thyroid
SH2D5 [SH2 domain containing 5]	−0.61	others	
CD74 [CD74 major histocompatibility complex]	−0.61	transmembrane receptor	
NDRG1 [N-myc downstream regulated 1]*	−0.62	enzyme (kinase)	E2
PRSS33 [protease, serine, 33]	−0.69	enzyme (peptidase)	
POP5 [processing of precursor 5]	−0.70	enzyme (nuclease)	
SSR2 [signal sequence receptor, beta]	−0.74	others	E2
ACADM [acyl-Coenzyme A dehydrogenase; MCAD]*	−0.80	enzyme (metabolic)	E2

*the zebrafish genes were subsequently validated using real-time PCR on a new batch of fish.

a average expression fold-change above control (log_2_ ratio) for 500 µg/L, 1500 µg/L and 4500 µg/L BPA (microarray data).

b refer to Table S6 for references reporting on the endocrine regulation of the genes.

c up-regulated at 500 µg/L but down-regulated at 1500 µg/L and 4500 µg/L BPA (microarray data).

d down-regulated at 500 µg/L but up-regulated at 1500 µg/L and 4500 µg/L BPA (microarray data).

### Inference of molecular mechanism underlying BPA early-life exposure toxicity via network connectivity analysis

**Figure 7:**
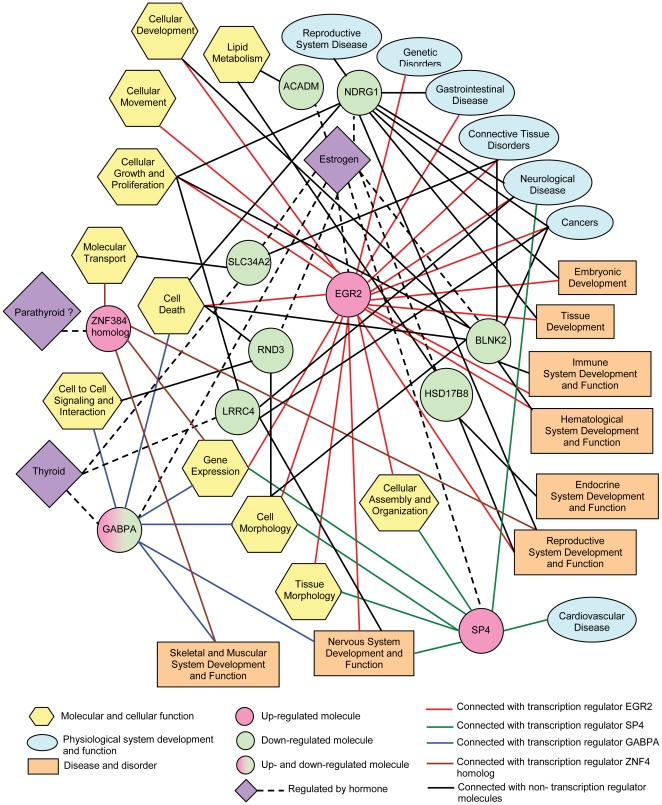
Real-time PCR validation of selected down-regulated zebrafish genes. Using samples from a separate validation experiment (n = 5–6 biological replicates; each replicate consist of five pooled larvae), selected down-regulated genes were validated by real-time PCR and compared with corresponding microarray data. Axis titles on the upper left histogram and figure legends are applicable to all histograms.

We submitted the 38 human homologs that were deregulated in all the treatment groups for Ingenuity Pathway Analysis™ and the algorithm identified 23 of the homologs to be significantly associated with the analysis. Using only the known endocrine-regulated homologs and their associations with the functional subcategories (see **Table S7**), a network was generated to visualize their connectivity and to further understand how these targeted genes could have associated with BPA early-life exposure toxicity (**Figure 7**). Among the four transcription regulators, EGR2 connected to 19 functional subcategories, has the highest connectivity involving various cellular functions (cell death, cellular growth and proliferation, cellular organization and assembly, cellular movement and development, cell and tissue morphology) in several physiological tissues/systems (nervous, hematological, immune and reproductive systems and cancer cells) suggesting vast biological role/impact of this deregulated gene (see references within **Table S7**). Transcription regulator SP4 is connected to 7 subcategories involving cellular organization and assembly, cell and tissue morphology which are associated with nervous system development (patterning and remodeling of neurons) and neurological degenerative disease (**Figure 7**; **Table S7**). SP4 is also required for development of the cardiac conduction system hence associated with cardiovascular disease (sinus bradycardia and ventricular tachycardia). The up-regulation of both *EGR2* and *SP4* by estrogen had been shown to be mediated by phosphatidylinositol 3-phosphate (PI3K) in a non-genomic manner [50]. More recent reports indicated that BPA could also exert its estrogenic activity via non-genomic mechanism [47]–[49]. Taken together, this suggests that the up-regulation of these two transcription regulators may be induced by BPA via PI3K in a non-genomic manner. Transcription regulator GABPA, connected to 6 subcategories involved in cell death, cell morphology and cell to cell signaling and interaction, is required for structural formation and function of neuromuscular junctions in skeletal muscle hence its association with the development and function of nervous, skeletal and muscular systems (**Figure 4**; **Table S7**). Estradiol treatment has been reported to down-regulate *GABPA* [51] and the down-regulation of zebrafish *gabpa* at 1500 ug/L and 4500 ug/L BPA observed in our study may be attributed to the estrogenic activity of BPA although the actual mechanism of this down-regulation is still not clear. Transcription regulator ZNF384 homolog is a nucleocytoplasmic shuttling protein which regulates the expression of collagen and matrix metalloproteinases important for bone development and is also required for spermatogenesis, hence its association with the development and function of skeletal, muscular and reproductive systems (**Figure 4**; **Table S7**). Estradiol and/or BPA are known to affect spermatogenesis [52] and bone development [53] although it is not clear how ZNF384 is up-regulated by BPA in these processes which therefore warrant further focused investigation.

Among the ‘Molecular and Cellular Function’ subcategories, ‘Cell Death’ (connected with GABPA, EGR2, NDRG1, RND3, BLNK) and ‘Cell Morphology’ (connected with EGR2, NDRG1, RND3, BLNK, and SP4) had the highest (5) connectivity (**Figure 4**). Based on the known developmental functions of these genes, cell death and cell morphological changes may be largely occurring in the nervous, blood/immune and reproductive systems. Nervous system (connected with EGR2, GABPA, LRRC4 and SP4) and reproductive system (connected with EGR2, HSD17B8, NDRG1 and ZNF384 homolog), had the highest (4) connectivity among the ‘physiological system development and function’ category suggesting that these organ-systems are likely to be impacted developmentally by BPA. Cancer (connected with EGR2, LRRC4, NDRG1 and BLNK2) and neurological disease (connected with EGR2, LRRC4, NDRG1 and SP4) has the highest (4) connectivity suggesting increased risk or susceptibility to these conditions in BPA-treated developing fish.

Taken together, the analysis reveals that BPA early-life exposure toxicity involved deregulation of endocrine-regulated genes with multiple roles in gene expression, signaling, transportation, enzymatic functions that regulates cellular functions (e.g. cell death, cell morphology, and cell differentiation) and development of several organ systems (nervous, reproductive and blood/immune systems being most vulnerable). This in turn may lead to increased risk of related diseases/disorders. Several of these affected physiological systems (nervous, cardiovascular, blood, reproductive, skeletal and muscular) and potential health impact have been either demonstrated *in vitro* and/or in rodents exposed to BPA or more potent estrogenic compounds [7]–[9], [47], [54], [55]. Specifically, it has been established that prenatal and/or neonatal exposure to low doses of BPA causes organizational changes in the reproductive tissues (prostate, breast, testis, mammary glands), body size, brain structure and chemistry, and behavior of laboratory animals that persist to adulthood, long after the period of exposure has ended [7], [8]. There is also evidence that BPA modulates the immune system [7] and may be associated with increased cancer of the hematopoietic and reproductive systems [9].Therefore, our findings are in line with the evidence of BPA adverse effects in laboratory animal studies and the concerns expressed by the latest NTP-CERHR expert panel report [6] with regard to the effects of BPA on the nervous system and behavior as well as reproductive tissues, in fetuses, infants, and children that are expose to BPA. Even so, these are inferences made based on association hence conclusive links and roles of these genes with BPA and the phenotypes observed would require more focused study on these genes in rodent or even appropriate in vitro cell lines. It will be interesting to investigate if similar mode-of-action is also associated with the adverse effects on nervous, reproductive and blood/immune systems reported in rodent studies [7], [8].

### Conclusion and Future Studies

The present study provided molecular insights into BPA early-life exposure toxicity in zebrafish involving disruption of endocrine-regulated gene expression and other signaling pathways that are known to play important roles in the development of tissue-organs, especially nervous, reproductive, cardiovascular, skeletal-muscular and blood systems. The findings corroborated with those reported in rodent studies in terms of BPA exerting endocrine disrupting effects and BPA affected organ-tissues, especially on the nervous, reproductive and blood systems. Even so, direct comparison with rodent data is still difficult due to many differing factors (e.g. physiology, life stages, mode and duration of exposure). Future studies involving absorption, distribution, metabolism and excretion (ADME) profile of BPA in developing zebrafish may help to generate scaling factors that could facilitate comparison and *in vivo-in vivo* correlation between the zebrafish and rodent models, or perhaps even humans. Nevertheless, this study demonstrated the potential of using zebrafish as a model for toxicogenomic inference of early-life exposure toxicity through BPA-induced cumulative phenotype and transcriptome analyses. Our findings revealed that zebrafish toxicogenomics could capture molecular information involving genes and signaling pathways that are useful to infer affected development and function of physiological systems as well as potential health-risks. Even so, we must stress that judicial interpretation is still required and while corroboration with phenotypic data could strengthen the analysis, a focused study on related genes is still required to be conclusive. Nevertheless, from the perspective of toxicity and hazard inference during early screening of compounds, such toxicogenomic information coupled with phenotypic data could serve well to alert investigators of a compound's potential health liabilities and provide needed information to conduct focused studies to investigate its specific toxicity. Finally, through this study we were able to identify a set of endocrine-regulated genes operating during early-life of zebrafish that are sensitive to BPA perturbation. These genes can serve as biomarkers or targets for more focused investigations such as kinetic studies with multiple time-point samplings or for mechanistic studies. Since the approach presented here is applicable to other typical compounds, our findings will place zebrafish in a strategic position for toxicogenomics application in early-life toxicity testing and as hazard indicator of various compounds. This is significant in view that there are strong demands and advocates for using zebrafish as an alternative model for evaluating the toxicity of pharmaceuticals, industrial chemicals and effluents by regulators and industry.

## Materials and Methods

### Ethics statement

All experimental protocols were approved by Institutional Animal Care and Use Committee (IACUC) of National University of Singapore (Protocol 079/07).

### BPA exposure and fish sampling

Three types of experiments were performed in this study, i.e. general acute toxicity tests, microarray experiment and PCR biomarker validation experiment. Experimental procedures were performed within the guidelines of National University of Singapore's Institutional Animal Care and Use Committee (NUS-IACUC). Different batches of developing zebrafish were exposed to BPA (Sigma-Aldrich, USA) at nominal concentrations between 50–5000 µg/L with 0.05% ethanol (vehicle) in egg water. In the acute toxicity test and PCR biomarker validation experiments, developing zebrafish were exposed to 50, 100, 500, 1500 and 4500 µg/L BPA for 7 days from 3 hour post-fertilization onwards while in the microarray experiment, developing zebrafish were exposed to 500, 1500 and 4500 µg/L BPA for the same duration. In experiments involving transgenic fish, exposure were carried out at 50, 500, and 5000 µg/L BPA for 5 days from 3 hour post-fertilization onwards. All control fish were maintained in egg water with 0.05% ethanol (vehicle). The developing zebrafish were maintained at an ambient temperature of about 26±1°C in a static condition and fresh media were renewed daily. At the end of the experiments, fish were examined for various phenotypic end-points, or snap-frozen in liquid nitrogen and stored at −80°C for total RNA extraction for microarray and PCR biomarker validation experiments.

### Total RNA extraction

Total RNA was extracted from 50 pooled larvae for each group using Trizol reagent (Invitrogen, USA) according to the manufacturer's instructions. Reference RNA for microarray hybridization was obtained by pooling zebrafish whole adult male and female total RNA at 9∶1 ratio. The integrity of RNA samples was verified by gel electrophoresis, and the concentrations were determined by UV spectrophotometer.

### Microarray hybridization

The arrays contained 22K oligonucleotide probes where 16.4K probes were designed by Compugen (USA) and the remaining probes were in-house designed by the bioinformatics group in Genome Institute of Singapore (GIS). The probes were resuspended in 3× SSC at 20 µM concentration and spotted onto in-house poly-L-lysine-coated microscope slides using a custom-built DNA microarrayer in GIS.

A common reference design is used where equal amount of total RNA from control or BPA-treated groups were labeled with Cy5 and co-hybridized with equal amount of common pooled reference RNA labeled with Cy3 for each array. Hence, for fluorescence labeling of cDNAs, 10 µg of total RNA from the reference and samples (treated and control groups) were reverse transcribed in the presence of dNTPs mixed with Aminoallyl-dUTP (Sigma-Aldrich, USA) followed by coupling with mono-functional NHS-ester Cy3 and Cy5 dyes (Amersham, USA), respectively. The respective paired Cy5- and Cy3-labeled cDNAs were pooled, concentrated, and resuspended in DIG EasyHyb (Roche Applied Science) buffer for hybridization on a single array at 42°C for 16 h in a hybridization chamber (MAUI, USA). After hybridization, the array-slides were washed in a series of washing solutions (2× SSC with 0.1% SDS, 1× SSC with 0.1% SDS, 0.2× SSC and 0.05× SSC; 30 sec each), dried using low-speed centrifugation, and scanned for fluorescence detection.

### Data acquisition, normalization and statistical filtering

The arrays were scanned using the GenePix 4000B microarray scanner (Axon Instruments, USA) and the generated images with their fluorescence signal intensities were analyzed using GenePix Pro 4.0 image analysis software (Axon Instruments, USA). Only gene features that were not flagged were extracted and subjected to Lowess normalization for further analyses. The microarray raw data have been formatted to be compliant with MIAME standard and has been submitted to GEO database (GEO Number: GSE22634).

Since a common reference design was used in our two-color array experiment, it allows for direct comparison of expression data between the treatment and control groups (*n* = 4 replicates). An initial One-Way ANOVA was applied on the Lowess-normalized data to narrow down genes that were differentially expressed and the resulting *P*-value of each gene was adjusted for Benjamini and Hochberg False Discovery Rate (FDR). To further obtain significant probes in each of the treatment group compared with the control group, Student's t-test was applied between the treatment and the control groups on the 20% FDR-selected dataset. Estimates of fold change were calculated, and genes with *P*-value<0.05, were considered for further analysis.

### Functional mapping and inference via knowledge-based data mining

To obtain insights into the biological functions and for health-risk inferences in humans, the zebrafish genes were mapped to their corresponding human homologs using the GIS Zebrafish Microarray Annotation Database (http://123.136.65.67/) as previously described [24]. National Center for Biotechnology Information (NCBI, USA) HomoloGene and UniGene databases were used for human homology mapping of the zebrafish genes. HomoloGene allows for detection of putative homologs among the annotated genes of several eukaryotic genomes and has links to UniGene clusters established by *tblastn* search of the UniGene database. The latest UniGene and HomoloGene Build files were downloaded from the following sites ftp://ftp.ncbi.nih.gov/repository/UniGene/Danio_rerio/ and ftp://ftp.ncbi.nlm.nih.gov/pub/HomoloGene/ , respectively. A PERL script was written to map all zebrafish UniGene clusters to human UniGene clusters that are identified as homologs of each other by the HomoloGene database. Another PERL script was written to enable automated mapping of GenBank Identifiers (GenBank Accession Number) of the zebrafish probes on the array to their respective UniGene cluster which are then mapped to human UniGene cluster(s) that has been identified as homolog(s) by HomoloGene database. This automated procedure is part of the Genome Institute of Singapore Zebrafish Microarray Annotation Database and is updated periodically from several resource databases.

The human homologs of the zebrafish genes from the filtered dataset were used to mine the human database via Ingenuity Pathway Analysis™ software (www.ingenuity.com). The software identifies three primary categories of functions, (i) molecular and cellular function, (ii) physiological system development and function, and (iii) disease and disorder, whereby each category is further subdivided into multiple levels of functional subcategories that are significantly enriched with human homologs of zebrafish genes from the input dataset. A right-tailed Fisher's Exact test was used to calculate *P*-values in determining the probability that each functional category enriched with the human homologs hence its association with the dataset is due to chance alone. *P*<0.05 is considered significant by the algorithm suggesting that the association is non-random. A network limited to the endocrine-regulated genes that are significant in all the treatment groups is generated by maximizing the specific connectivity of the mapped human homologs with other enriched functional subcategories.

### Biomarker validation using real-time PCR

To validate the microarray data, two independent validation experiments were carried out from new batches of developing zebrafish similar to the earlier acute toxicity experiment (*n* = 5–6 biological replicates; each biological replicate consists of five pooled larvae). Equal amounts of total RNA samples were reverse transcribed to cDNA (Invitrogen, USA). The cDNA samples were used for quantitative real-time PCR analysis, performed using the Lightcycler system (Roche Applied Science) with Lightcycler-FastStart DNA Master SYBR Green 1 (Roche Applied Science) according to the manufacturer's instructions. Statistical comparison of the relative mean expression level for each gene between test and control groups was performed using Student's T-test and *P*<0.05 is considered significant. Estimate of fold change (log_2_) between each treatment group and control group were presented.

## Supporting Information

Figure S1 **Inhibition of axon growth and branching was observed in** ***Tg(Nkx2.2a-mEGFP)*** **line treated with 17-beta estradiol (E2 at 10, 50, 100 µg/L) but not with mefenamic acid (MA at 10, 50, 100 µg/L) when compared to control group.**
(TIF)

Figure S2 **Real-time PCR validation of the six genes (** ***ncl1, apoeb, mdm1, mycl1b, sp4, U1SNRNPBP*** **homolog) using samples from wildtype developing zebrafish treated with 17-beta estradiol [E2 at 10 µg/L (36.7 nM), 50 µg/L (183.5 nM), 100 ug/L (367.1 nM) ], mefenamic acid [MA at 10 µg/L (41.4 nM), 50 µg/L (207.0 nM), 100 µg/L(414.4 nM)] and Bisphenol A [BPA at 50 µg/L (207.0 nM), 100 µg/L(438.0 nM)].** Numbers in the cell indicate mean expression levels (log_2_ fold-change) above control group (n = 5–6).
(TIF)

Table S1 **Information of zebrafish transgenic lines used in present study.**
(PDF)

Table S2 **Selected top functional subcategories of ‘Molecular and cellular function’ that are significantly enriched with human homologs of zebrafish genes deregulated by 500 µg/L, 1500 µg/L and 4500 µg/L of BPA.**
(PDF)

Table S3 **Selected top functional subcategories of ‘Physiological system development and function’ that are significantly enriched with human homologs of zebrafish genes deregulated by 500 µg/L, 1500 µg/L and 4500 µg/L of BPA.**
(PDF)

Table S4 **Selected top functional subcategories of ‘Disease and disorder’ that are significantly enriched with human homologs of zebrafish genes deregulated by 500 µg/L, 1500 µg/L and 4500 µg/L of BPA.**
(PDF)

Table S5 **Selected top functional subcategories of ‘Canonical Pathways’ that are significantly enriched with human homologs of zebrafish genes deregulated by 500 µg/L, 1500 µg/L and 4500 µg/L of BPA.**
(PDF)

Table S6 **Human homolog of zebrafish genes that were significantly deregulated in all three BPA exposed groups (500 µg/L, 1500 µg/L and 4500 µg/L).**
(PDF)

Table S7 **Selected functional subcategories that are significantly (Fisher's Exact Test P<0.05) enriched with human homologs of zebrafish genes deregulated in all BPA treatment groups (500 µg/L, 1500 µg/L and 4500 µg/L).**
(PDF)
