# Supplementary figures and images for: Toxicogenomic and Phenotypic Analyses of Bisphenol-A Early-Life Exposure Toxicity in Zebrafish
Source: PLoS One. 2011 Dec 14;6(12):e28273. doi: 10.1371/journal.pone.0028273 (PMC3237442; doi:10.1371/journal.pone.0028273)

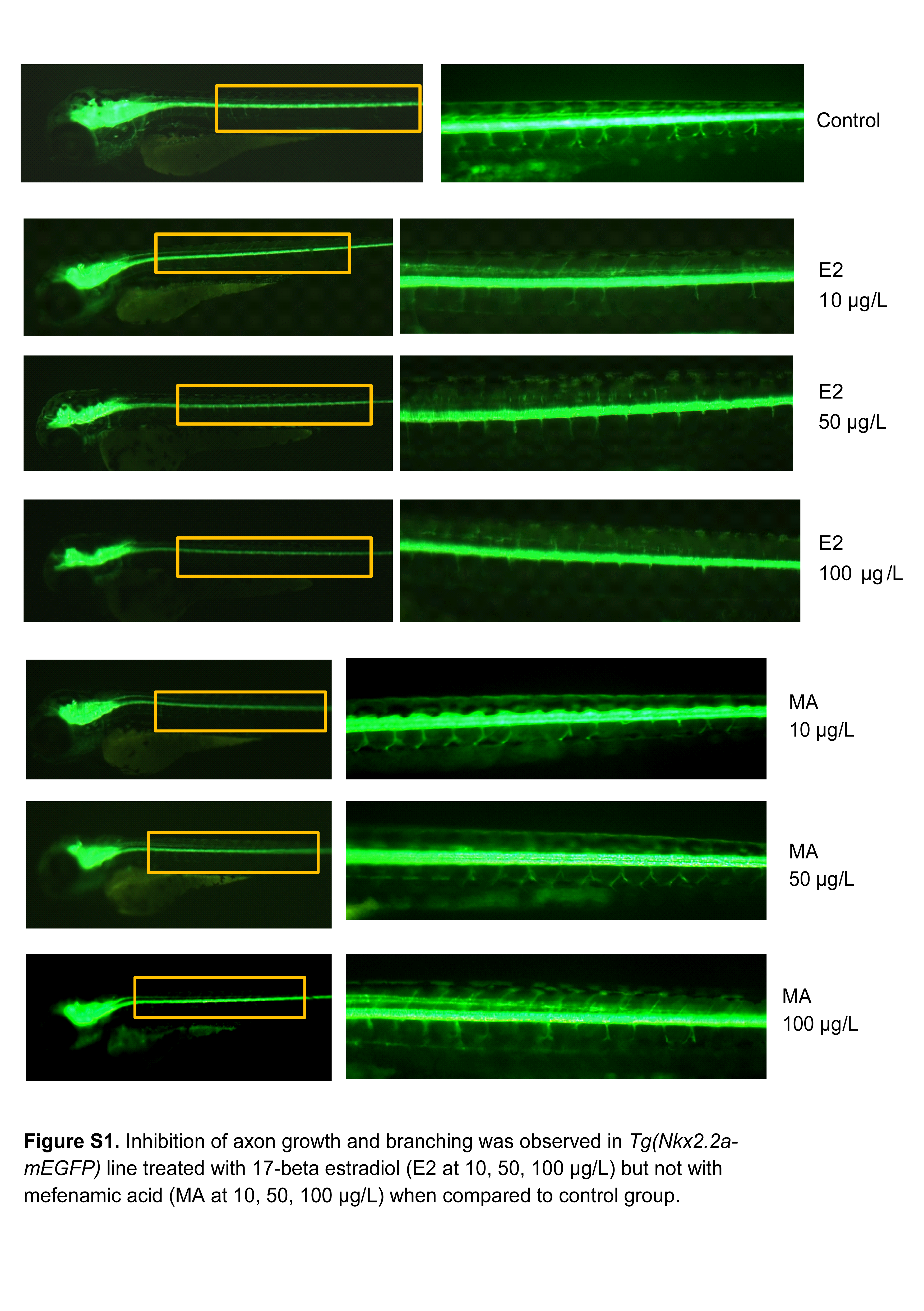

Supplement: Figure S1 — Inhibition of axon growth and branching was observed in Tg(Nkx2.2a-mEGFP) line treated with 17-beta estradiol (E2 at 10, 50, 100 µg/L) but not with mefenamic acid (MA at 10, 50, 100 µg/L) when compared to control group. (TIF) [file pone.0028273.s001.tif]

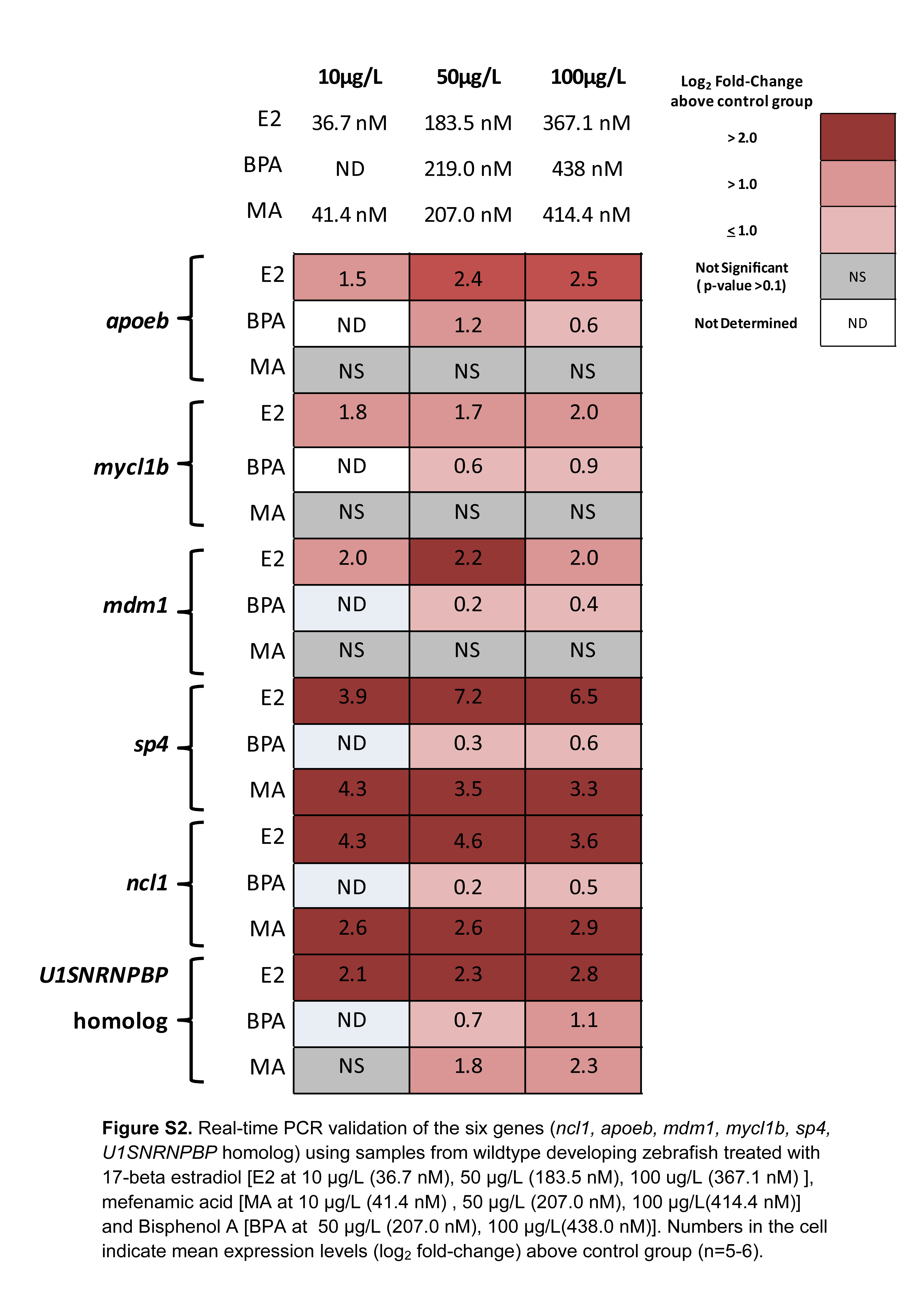

Supplement: Figure S2 — Real-time PCR validation of the six genes ( ncl1, apoeb, mdm1, mycl1b, sp4, U1SNRNPBP homolog) using samples from wildtype developing zebrafish treated with 17-beta estradiol [E2 at 10 µg/L (36.7 nM), 50 µg/L (183.5 nM), 100 ug/L (367.1 nM) ], mefenamic acid [MA at 10 µg/L (41.4 nM), 50 µg/L (207.0 nM), 100 µg/L(414.4 nM)] and Bisphenol A [BPA at 50 µg/L (207.0 nM), 100 µg/L(438.0 nM)]. Numbers in the cell indicate mean expression levels (log2 fold-change) above control group (n = 5–6). (TIF) [file pone.0028273.s002.tif]
