# Supplementary material for: Toxicogenomic and Phenotypic Analyses of Bisphenol-A Early-Life Exposure Toxicity in Zebrafish
Source: PLoS One. 2011 Dec 14;6(12):e28273. doi: 10.1371/journal.pone.0028273 (PMC3237442; doi:10.1371/journal.pone.0028273)
Supplement: Table S1 — Information of zebrafish transgenic lines used in present study. (PDF) [file pone.0028273.s003.pdf]

Table S1. Information of zebrafish transgenic lines used in present study.

Transgenic line [Description of green fluorescence protein expression]	Description of green fluorescence protein expression	Reference
**Tg(flia:EGFP)**	Blood circulatory system	Lawson ND, Weinstein BM. (2002). In vivo imaging of embryonic vascular development using transgenic zebrafish. Dev Biol. 248(2):307-18.
**Tg(nkx2.2a:mEGFP)**	Axon, motor neuron, myelin assembly, Schwann cell	Kucenas S, Snell H, Appel B. (2008). nkx2.2a promotes specification and differentiation of a myelinating subset of oligodendrocyte lineage cells in zebrafish. Neuron Glia Biol. 4(2):71-81.
**ET(krt8:EGFP)sqet20 (Enhancer Trap 20)**	Mantle cells of neuromasts of the lateral line	Parinov S, Kondrichin I, Korzh V, Emelyanov A. (2004). Tol2 transposon-mediated enhancer trap to identify developmentally regulated zebrafish genes in vivo. Dev. Dynam, 231:449-459.
