# Supplementary material for: Toxicogenomic and Phenotypic Analyses of Bisphenol-A Early-Life Exposure Toxicity in Zebrafish
Source: PLoS One. 2011 Dec 14;6(12):e28273. doi: 10.1371/journal.pone.0028273 (PMC3237442; doi:10.1371/journal.pone.0028273)
Supplement: Table S2 — Selected top functional subcategories of ‘Molecular and cellular function’ that are significantly enriched with human homologs of zebrafish genes deregulated by 500 µg/L, 1500 µg/L and 4500 µg/L of BPA. (PDF) [file pone.0028273.s004.pdf]

Table S2. Selected top functional subcategories of 'Molecular and cellular function' that are significantly enriched with human homologs of zebrafish genes deregulated by 500 µg/L, 1500 µg/L and 4500 µg/L of BPA. The data was generated using Ingenuity Pathway Analysis™ software and only subcategories with P <0.05 (Fisher's Exact Test) and having greater than 5% of homologs enriched in all three treatment groups are listed.

Functional Subcategories	Percentage (no. molecules enriched / no. molecules used in analysis)	Molecules (human homologs of zebrafish genes) used in the analysis
**Cell Death**		
BPA_500µg	39.6% (40/101)	BLNK,SRPX,PA2G4,AXIN1,BDNF,FGF8,FIP1L1,MYH11,CD74,SCYE1,TGM2,MYLK,CSNK1E,PHOX2A,HK1,CASP6,CD2AP,EGR2,CAMK2D,PARK7,PIK3CG,ZHX2,TTC5,PPP3CA,RUNX3,E2F4,TJP2,NDRG1,FEM1B,IFT57,DPF2,GABBR1,CDH2,CD68,GAB1,RND3,SYK,RHOA,GABPA,SPO11
BPA_1500µg	43.5% (73/168)	AXIN1,PTHLH,CDC7,HRAS,CDK9,SCYE1,LRPAP1,PTK2,CAMK2D,CDK8,EGR2,ZHX2,PIK3CG,PRKAA1,IRS2,CCNL2,E2F4,NDRG1,TFDP1,FEM1B,IFT57,CREBBP,COL2A1,TCF3,RNF17,ACVR1B,PFKM,HSPA8,PRPH2,TGM1,PPP1R13L,GAB1,CDH5,RND3,SYK,RTN4,SPO11,BLNK,POU3F1,APOE,CUL5,SRPX,PPP2R2A,BDNF,PCDH21,HYOU1,FGF8,FIP1L1,CD74,CSNK1E,CASP6,AQP3,HAND2,YES1,AKT1,CLDN4,EFNB1,SORT1,PPP3CA,GALNT10,NUDCD3,GRIN1,KHK,PTP4A2,VPS33A,DEDD2,CCDC6,ROCK1,GRAP2,GABPA,HOXB9,CYP2J2,ERCC5
BPA_4500µg	28.8% (36/125)	APOE,APOB,SRPX,PA2G4,AXIN1,HYOU1,CDC7,HRAS,FIP1L1,SETX,MYH11,PTK2,EGR2,PARK7,PTPRO,TGFB2,TTC5,PIK3R2,PPP3CA,ITGB5,CCNL2,PPARD,RPA1,TCF3,ACVR1B,IGF2,TGM1,PPP1R13L,NRCAM,BHLHB2,RTN4,GABPA,CTH,CFLAR,CYR61,TNFRSF19
**Cell Morphology**		
BPA_500µg	22.7% (18/101)	NCKIPSD,MYCL1,ZNF384,AXIN1,BDNF,USF2,FGF8,FIP1L1,MYH11,CD74,MYLK,CASP6,CD2AP,BCKDHA,PIK3CG,E2F4,YWHAG,DDEF1,SP4,SEC23A,CDH2,SDCBP,GAB1,RND3,RHOA,SYPL2,GABPA,CPEB1
BPA_1500µg	22.0% (27/168)	APOE,GEM,MYCL1,ZNF384,BDNF,AXIN1,FGF8,HRAS,FIP1L1,DNAJC3,LRPAP1,FRAG1,COL1A2,PTK2,AQP3,YES1,AKT1,PIK3CG,BCKDHA,EFNB1,ZHX2,DENND2D,E2F4,MLLT10,TFDP1,RRAD,CREBBP,SLIT2,RNF17,TCF3,CCDC6,SP4,ROCK1,PPP1R13L,RND3,GAB1,RTN4
BPA_4500µg	27.2% (29/125)	APOE,GEM,EPS8,NCDN,STMN2,ZNF384,AXIN1,EXT1,HRAS,FIP1L1,MYH11,FRAG1,PTK2,EFNB1,TGFB2,GFAP,DENND2D,YWHAG,TFDP1,RRAD,SLIT2,RNF17,TCF3,SP4,ROCK1,SDCBP,PPP1R13L,RND3,NRCAM,RTN4,GABPA,GOLGA5,CYR61,TNFRSF19

Cellular Assembly and Organization

BPA_500µg	16.8% (17/101)	NCKIPSD,DDEF1,BDNF,MYH11,ACTG1,SP4,CASP6,CDH2,CD2AP,TIAM2,CLDN4,GAB1,SYK,RHOA,SPO11,PPP3CA,ACTA1
BPA_1500µg	21.4% (36/168)	POU3F1,APOE,GEM,NCKIPSD,BDNF,CDC7,PCDH21,RAN,HRAS,POU3F2,LRPAP1,PTK2,DYNC1LI1,YES1,AKT1,EFNB1,GFAP,SORT1,CAV2,PPP3CA,GRIN1,NDRG1,RRAD,COL2A1,EXOC7,SLIT2,ACTG1,SP4,ROCK1,TIAM2,GAB1,NDE1,HELLS,SYK,RTN4,SPO11
BPA_4500µg	30.4% (38/125)	APOE,MYH6,EPS8,GEM,STMN2,DIAPH3,CDC7,RAN,EXT1,HEXA,HRAS,PTK2,PTPRO,EFNB1,MID1IP1,MAP1A,GFAP,SORT1,CAV2,PPP3CA,ITGB5,NDRG1,RRAD,PPARD,RPA1,SLIT2,ACTG1,SP4,ROCK1,NRCAM,HELLS,RTN4,GOLGA5,CAPN2,C10ORF119,CYR61,TNFRSF19,VIL1

Cellular Movement

BPA_500µg	22.8% (23/101)	RUNX3,DDEF1,BDNF,RAB35,FGF8,CCNB2,MYH11,SCYE1,TGM2,MYLK,TSPAN3,CDH2,CD2AP,EGR2,SDCBP,TIAM2,RND3,GAB1,CLDN4,PIK3CG,RHOA,SYK,CYSLTR1
BPA_1500µg	17.9% (30/168)	APOE,CUL5,GEM,BDNF,PTHLH,FGF8,HRAS,POU3F2,SCYE1,LRPAP1,FGF13,PTK2,EGR2,AKT1,CLDN4,EFNB1,PIK3CG,SORT1,SERPINA1,MLLT10,SLIT2,TCF3,ROCK1,TIAM2,RND3,CDH5,GAB1,NDE1,SYK,CYP2J2
BPA_4500µg	19.2% (24/125)	GEM,EPS8,HRAS,MYH11,SLIT2,TCF3,ROCK1,PTK2,IGF2,EGR2,SDCBP,RND3,NRCAM,JAM3,PTPRO,EFNB1,TGFB2,CYSLTR1,CAPN2,SERPINA1,PIK3R2,TBXAS1,VIL1,ITGB5

Small Molecule Biochemistry

BPA_500µg	28.7% (29/101)	SLC27A2,BDNF,USF2,MYLK,TGM2,HK1,PRPS1,CAMK2D,PARK7,PIK3CG,BCKDHA,SLC20A1,AFMID,CHERP,PGM1,GABBR1,SERINC1,SLC34A2,UMPS,EBP,RND3,GAB1,RHOA,SYK,SYPL2,ACAT1,NUCB2,CYSLTR1,SLC27A1
BPA_1500µg	14.9% (25/168)	APOE,FOXA1,BDNF,PPP1R3C,PTHLH,HRAS,CDK9,LRPAP1,PTK2,AKT1,CDK8,CHIT1,KARS,PRKAA1,IRS2,SERPINA1,NSDHL,RRAD,UPP1,RPIA,PFKM,TGM1,SLC27A1,TBXAS1,NPC2
BPA_4500µg	20.8% (26/125)	APOE,APOB,PPP1R3C,PKD2,HRAS,HEXA,ADIPOR1,PTK2,CAMK2D,PARK7,KARS,OXCT1,SERPINA1,GUCA1A,RRAD,PPARD,UPP1,CHERP,PGM1,UMPS,IGF2,TGM1,CYSLTR1,NUCB2,CTH,TBXAS1

Cellular Development

BPA_500µg	14.9% (15/101)	BLNK,RUNX3,E2F4,CDC2L5,AXIN1,BDNF,FGF8,MYH11,CD74,EGR2,SOX3,RHOA,SYK,PIK3CG,PITX3
BPA_1500µg	16.1% (27/168)	BLNK,POU3F1,BDNF,AXIN1,PTHLH,FGF8,CDC7,HRAS,DNAJC3,POU3F2,NKX2-3,CD74,PTK2,HAND2,EGR2,AKT1,PIK3CG,IRS2,PPP3CA,DENND2D,E2F4,MLLT10,SLIT2,TCF3,SYK,COL9A2,SPO11
BPA_4500µg	32.0% (40/125)	BLNK,MYH6,PA2G4,STMN2,NID2,AXIN1,CDC7,HRAS,SFPQ,EXT1,MYH11,CD74,SPRED2,NID1,PTK2,EGR2,TGFB2,ACADM,ITGB5,DENND2D,YWHAG,NDRG1,STK39,PPARD,MEOX1,SNRK,SLIT2,RNF17,TCF3,ACVR1B,IGF2,PPP1R13L,TGM1,HELLS,BHLHB2,EHMT2,SF3B4,TOX,DYRK1A,FNDC3A
**Cell Cycle**		
BPA_500µg	9.9% (10/101)	BLNK,CD2AP,E2F4,GAB1,MAD2L1BP,RHOA,RAB35,MRE11A,SPO11,SCYE1
BPA_1500µg	22.0% (37/168)	BLNK,APOE,CUL5,PPP2R2A,PTHLH,RAN,CDC7,FGF8,HRAS,NUP214,CDK9,SCYE1,PTK2,DYNC1LI1,CAMK2D,AKT1,CDK8,MAD2L1BP,IRS2,MAP9,PPP3CA,NUDCD3,E2F4,TFDP1,NDRG1,RRAD,CREBBP,TCF3,ACVR1B,HSPA8,ANAPC4,C10ORF46,GAB1,NDE1,HELLS,SYK,SPO11
BPA_4500µg	11.2% (14/125)	BLNK,YWHAG,NDRG1,TFDP1,CDC7,RAN,HRAS,RPA1,PTK2,IGF2,HELLS,PTPRO,BHLHB2,CYR61
**Gene Expression**		
BPA_500µg	29.7% (30/101)	ECSIT,BLNK,ZNF384,PA2G4,BDNF,AXIN1,USF2,MYH11,SET,CEBPG,CSNK1E,PHOX2A,EGR2,CAMK2D,BCKDHA,ZHX2,SIN3B,TTC5,PPP3CA,SLC20A1,RUNX3,E2F4,SP4,SUPT16H,RHOA,SYK,GABPA,TFB2M,PITX3,DYRK1A
BPA_1500µg	26.7% (28/168)	NR6A1,ZNF384,AXIN1,BDNF,FOXA1,HRAS,POU3F2,CDK9,CEBPG,FGF13,HAND2,AKT1,EGR2,ZHX2,ALAS1,PPP3CA,E2F4,TFDP1,CREBBP,PFDN5,TCF3,RNF17,ACVR1B,ROCK1,EAPP,GRAP2,SYK,GABPA
BPA_4500µg	8.0% (10/125)	ROCK1,IGF2,EGR2,DNMT3A,HELLS,TGFB2,HBE1,HRAS,RNF17,TCF3
**Cellular Growth and Proliferation**		
BPA_500µg	7.9% (8/101)	RUNX3,CDH2,CDC2L5,AXIN1,BDNF,FGF8,CYSLTR1,PPP3CA
BPA_1500µg	13.1% (22/168)	BDNF,PTHLH,CDC7,FGF8,DNAJC3,HRAS,NKX2-3,SLIT2,TCF3,FGF13,PTK2,EAPP,AKT1,CDH5,GRAP2,EFNB1,PIK3CG,SYK,CYP2J2,COL9A2,DPH1,PPP3CA

BPA_4500µg	12.0% (15/125)	AXIN1,CDC7,HRAS,SLIT2,TCF3,IGF2,EGR2,PTPRO,EFNB1,CYSLTR1,CYR61,DPH1,TOX,ITGB5,PPP3CA

Cell-To-Cell Signaling and Interaction

BPA_500µg	15.8% (16/101)	YWHAG,NCKIPSD,TJP2,BDNF,DDEF1,ADIPOR1,TGM2,CDH2,CLDN4,RND3,SYK,PIK3CG,RHOA,KCNJ1,GABPA,CPEB1
BPA_1500µg	9.5% (16/168)	APOE,GRIN1,GEM,BDNF,RRAD,PTHLH,HRAS,SLIT2,LRPAP1,PTK2,YES1,AKT1,CDH5,SYK,EFNB1,GABPA
BPA_4500µg	10.4% (13/125)	APOE,NCDN,YWHAG,GEM,RRAD,HRAS,PTK2,IGF2,RND3,JAM3,EFNB1,GABPA,ITGB5

Cellular Compromise

BPA_500µg	7.9% (8/101)	CASP6,CDH2,NDRG1,BDNF,SUPT16H,RHOA,SET,CEBPG
BPA_1500µg	7.7% (13/168)	DENND2D,GEM,NDRG1,RRAD,BDNF,HRAS,SET,CEBPG,ROCK1,PTK2,TGM1,EFNB1,MID1IP1
BPA_4500µg	10.4% (13/125)	DENND2D,APOE,GEM,STMN2,NDRG1,RRAD,HRAS,ROCK1,PTK2,TGM1,EFNB1,MAP1A,MID1IP1

Cellular Function and Maintenance

BPA_500µg	9.9% (10/101)	NCKIPSD,BDNF,SYK,RHOA,SYPL2,CHERP,KCNJ1,PGM1,NUCB2,ACTG1
BPA_1500µg	6.% (10/168)	PTK2,APOE,YES1,NCKIPSD,AKT1,CDH5,BDNF,SYK,TBXAS1,ACTG1
BPA_4500µg	10.4% (13/125)	APOE,MYH6,PKD2 CHERP,PGM1,ACTG1,PTK2,IGF2,PTPRO,EFNB1,NUCB2,CFLAR,TBXAS1

Molecular Transport

BPA_500µg	8.9% (9/101)	SLC20A1,PARK7,BDNF,USF2,KCNJ1,ACAT1,CYSLTR1,SLC27A1,SLC34A2
BPA_1500µg	10.1% (17/168)	APOE,ZNF384,KPNA6,RRAD,FOXA1,PPP1R3C,PTHLH,RAN,NUP214,HRAS,LRPAP1,ROCK1,AKT1,SORT1,SLC27A1,IRS 2,PPP3CA

BPA_4500µg	13.6% (17/125)	SLC20A1,APOE,APOB,ZNF384,PPARD,RRAD,PPP1R3C,RAN,HEXA,HRAS,SLC34A2,ADIPOR1,ROCK1,PTK2,PARK7,CYSLTR1,SORT1
**Lipid Metabolism**		
BPA_500µg	7.9% (8/101)	EBP,SLC27A2,BDNF,SYK,PIK3CG,ACAT1,CYSLTR1,SLC27A1
BPA_1500µg	8.9% (15/168)	APOE,NSDHL,BDNF,RRAD,FOXA1,PPP1R3C,PTHLH,HRAS,LRPAP1,AKT1,IRS2,SERPINA1,SLC27A1,TBXAS1,NPC2
BPA_4500µg	9.6% (12/125)	APOE,APOB,RRAD,PPARD,PPP1R3C,OXCT1,CYSLTR1,HRAS,HEXA,SERPINA1,TBXAS1,ADIPOR1
**DNA Replication, Recombination, and Repair**		
BPA_500µg	5.9% (6/101)	TGM2,RND3,RHOA,XRN2,MRE11A,SPO11
BPA_1500µg	5.4% (9/168)	DYNC1LI1,NDRG1,HELLS,NDE1,UPP1,CDC7,RAN,ERCC5,SPO11
BPA_4500µg	6.4% (8/125)	IGF2,NDRG1,DNMT3A,HELLS,XRN2,UPP1,CDC7,RAN
