# Supplementary material for: Toxicogenomic and Phenotypic Analyses of Bisphenol-A Early-Life Exposure Toxicity in Zebrafish
Source: PLoS One. 2011 Dec 14;6(12):e28273. doi: 10.1371/journal.pone.0028273 (PMC3237442; doi:10.1371/journal.pone.0028273)
Supplement: Table S3 — Selected top functional subcategories of ‘Physiological system development and function’ that are significantly enriched with human homologs of zebrafish genes deregulated by 500 µg/L, 1500 µg/L and 4500 µg/L of BPA. (PDF) [file pone.0028273.s005.pdf]

Table S3. Selected top functional subcategories of 'Physiological system development and function' that are significantly enriched with human homologs of zebrafish genes deregulated by 500 µg/L, 1500 µg/L and 4500 µg/L of BPA. The data was generated using Ingenuity Pathway Analysis™ software and only subcategories associated with specific organ-system with $P<0.05$ (Fisher's Exact Test) and having greater than 5% of homologs enriched in all three treatment groups are listed.

Functional Subcategories	Percentage (no. molecules enriched / no. molecules used in analysis)	Molecules (human homologs of zebrafish genes) used in the analysis
**Nervous System**		
BPA_500µg	17.8% (18/101)	RUNX3,YWHAG,BDNF,FGF8,SP4,PHOX2A,CDH2,TIAM2,EGR2,PARK7,RHOA,KCNJ1,CYSLTR1,GABPA,CPEB1,PPP3CA,PITX3,DYRK1A
BPA_1500µg	16.1% (27/168)	POU3F1,APOE,LHX5,CUL5,GEM,BDNF,FGF8,HRAS,POU3F2,LRPAP1,PTK2,EGR2,AKT1,EFNB1,GFAP,SORT1,PPP3CA,GRIN1,RRAD,CREBBP,SLIT2,SP4,ROCK1,TIAM2,NDE1,RTN4,GABPA
BPA_4500µg	23.2% (29/125)	APOE,GEM,NCDN,APOB,STMN2,ZNF384,HRAS,EXT1,MPPED2,PRPS1,EGR2,EFNB1,TGFB2,GFAP,ITGB5,PPP3CA,THBS4,YWHAG,RRAD,CHERP,SLIT2,SP4,ROCK1,IGF2,NRCAM,RTN4,GABPA,TNFRSF19,DYRK1A
**Skeletal and Muscular System**		
BPA_500µg	14.9% (15/101)	NCKIPSD,YWHAG,FGF8,MYH11,ACTG1,MYLK,TGM2,CDH2,CAMK2D,GAB1,RHOA,SYPL2,MYL4,GABPA,ACTA1
BPA_1500µg	6.0% (10/168)	PTK2,APOE,NCKIPSD,AKT1,GAB1,PTHLH,FGF8,GABPA,SLIT2,ACTG1
BPA_4500µg	12.0% (15/125)	APOE,MYH6,YWHAG,MEOX1,MYH11,SLIT2,ACTG1,SPRED2,PTK2,IGF2,HCN2,SP8,BHLHB2,TGFB2,GABPA
**Hematological System**		
BPA_500µg	13.9% (14/101)	BLNK,RUNX3,E2F4,CDC2L5,BDNF,MYH11,CD74,SCYE1,MYLK,EGR2,SYK,PIK3CG,RHOA,CYSLTR1
BPA_1500µg	9.5% (16/168)	BLNK,APOE,BDNF,PTHLH,HRAS,NKX2-3,CD74,TCF3,SCYE1,PTK2,AKT1,EGR2,GRAP2,SYK,EFNB1,PIK3CG

BPA_4500µg	6.4% (8/125)	PTK2,APOE,PTPRO,HRAS,CYSLTR1,CFLAR,TCF3,TOX
**Cardiovascular System**		
BPA_500µg	7.9% (8/101)	CDH2,CAMK2D,GAB1,BDNF,PIK3CG,RHOA,FGF8,SCYE1
BPA_1500µg	7.7% (13/168)	APOE,BDNF,PTHLH,HYOU1,FGF8,SCYE1,PTK2,AKT1,CAMK2D,CDH5,GAB1,EFNB1,CFB
BPA_4500µg	10.4% (13/125)	APOE,APOB,HYOU1,SPRED2,PTK2,IGF2,HCN2,EFNB1,CFB,RTN4,TGFB2,CYR61,CAV2
**Reproductive System**		
BPA_500µg	6.9% (7/101)	NDRG1,GAB1,AXIN1,USF2,SOX3,RHOA,SPO11
BPA_1500µg	6.5% (11/168)	PTK2,DENND2D,AQP3,TGM1,NDRG1,AXIN1,PTHLH,FGF8,HOXB9,IRS2,SPO11
BPA_4500µg	7.2% (9/125)	PTK2,DENND2D,IGF2,TGM1,NDRG1,EHMT2,HRAS,RNF17,FNDC3A
