# Supplementary material for: Toxicogenomic and Phenotypic Analyses of Bisphenol-A Early-Life Exposure Toxicity in Zebrafish
Source: PLoS One. 2011 Dec 14;6(12):e28273. doi: 10.1371/journal.pone.0028273 (PMC3237442; doi:10.1371/journal.pone.0028273)
Supplement: Table S4 — Selected top functional subcategories of ‘Disease and disorder’ that are significantly enriched with human homologs of zebrafish genes deregulated by 500 µg/L, 1500 µg/L and 4500 µg/L of BPA. (PDF) [file pone.0028273.s006.pdf]

Table S4. Selected top subcategories of 'Disease and disorder' that were significantly enriched with human homologs of zebrafish genes deregulated by 500 µg/L, 1500 µg/L and 4500 µg/L of BPA. The data was generated using Ingenuity Pathway Analysis™ software and only subcategories with *P* <0.05 and having greater 5% of homologs enriched in at least two treatment groups are listed here.

Description	P-value (Fisher Exact Test)	Percentage (no. molecules enriched / no. molecules used in analysis)	Molecules (human homologs of zebrafish genes) used in the analysis
**Cancer**			
BPA_500µg	5.89E-04 to 3.6E-02	50.5% (51/101)	SLC27A2,ZNF384,AXIN1,NDRG2,CALCOCO2,CCNB2,MYH11,SCYE1,SET,MYLK,CD2AP,PIK3CG,BCKDHA,E2F4,YWHAG,NDRG1,MFAP5,CDH2,EBP,SDCBP,GAB1,RND3,RHOA,SYK,BLNK,MYCL1,PA2G4,SRPX,BDNF,USF2,FGF8,FIP1L1,MRE11A,CD74,ACOT7,SERPIND1,CSNK1E,CLDN4,PARK7,MAD2L1BP,SSR2,PPP3CA,RUNX3,ETFA,DDEF1,GABBR1,SLC34A2,DNAJB9,ACTG1,ACAT1,CYSLTR1
BPA_1500µg	3.4E-04 to 2.45E-02	55.4% (93/168)	ZNF384,SLC27A2,AXIN1,KRT7,PTHLH,CDC7,RAN,DNAJC3,HRAS,CCNB2,KIAA0746,CDK9,LRPAP1,SCYE1,SET,FRAG1,PTK2,CAMK2D,CDK8,BCKDHA,PIK3CG,PRKAA1,IRS2,E2F4,NDRG1,TFDP1,MLLT10,RRAD,ACTR10,CREBBP,COL2A1,PFDN5,RNF17,TCF3,PFKM,HSPA8,EAPP,PPP1R13L,CDH5,GAB1,RND3,SYK,ALDOA,TBXAS1,VIL1,BLNK,APOE,GEM,MYCL1,SRPX,BDNF,NID2,PPP1R3C,FGF8,HYOU1,FIP1L1,NUP214,CD74,FGF13,CSNK1E,COL1A2,AQP3,YES1,AKT1,CLDN4,MAD2L1BP,PDCD2L,EFNB1,CFB,ALAS1,SSR2,SORT1,SERPINA1,GBP2,DPH1,PPP3CA,GRIN1,NUDCD3,SERTAD2,ANKRD9,PTP4A2,KHK,ETFA,UPP1,RQCD1,SLIT2,SLC34A2,DNAJB9,CCDC6,ACTG1,ROCK1,EIF4A1,CYP2J2
BPA_4500µg	2.6E-05 to 3.13E-02	66.4% (83/125)	EPS8,ZNF384,NDRG2,AXIN1,KRT7,DIAPH3,RAN,CDC7,SFPQ,HRAS,MYH11,FRAG1,PTK2,EGR2,PTPRO,TTC5,ITGB5,CCNL2,YWHAG,TFDP1,NDRG1,STK39,RRAD,MEOX1,RPA1,RNF17,TCF3,ACVR1B,FLCN,IGF2,TGM1,PPP1R13L,SDCBP,NRCAM,RND3,JAM3,BHLHB2,CALB2,RTN4,GOLGA5,CFLAR,CYR61,TBXAS1,VIL1,BLNK,APOE,GEM,SRPX,STMN2,PA2G4,NID2,MXRA8,PPP1R3C,PKD2,HYOU1,FIP1L1,EXT1,SETX,CD74,PROM1,SERPIND1,PARK7,EFNB1,TGFB2,CFB,SSR2,SORT1,SERPINA1,PIK3R2,DPH1,PPP3CA,ETFA,PPARD,UPP1,TUBA4A,SLC34A2,EPHA3,SLIT2,ACTG1,ROCK1,GABPA,CYSLTR1,DYRK1A
**Neurological Disease**			
BPA_500µg	9.59E-03 to 2.97E-02	9.9% (10/101)	TGM2,RUNX3,PRPS1,EGR2,NDRG1,PARK7,BDNF,FGF8,ACAT1,GABBR1
BPA_1500µg	3.4E-04 to 2.51E-02	10.7% (18/168)	GRIN1,POU3F1,APOE,GEM,MAGT1,BDNF,RRAD,VPS33A,CREBBP,HYOU1,FGF8,HRAS,SETX,LRPAP1,CASP6,SOX3,EFNB1,JARID1C

BPA_4500µg	2.48E-04 to 3.13E-02	12.8% (16/125)	APOE,APOB,GEM,NDRG1,RRAD,UPP1,HRAS,SETX,IGF2,PPP1R13L,EGR2,PARK7,EFNB1,SP8,GFAP,CFLAR
**Connective Tissue Disorders**			
BPA_500µg	1E-02 to1.99E-02	15.8% (16/101)	BLNK,RUNX3,BDNF,FIP1L1,SLC34A2,CD74,ADIPOR1,TGM2,EBP,EGR2,PRPS1,CD68,GAB1,RHOA,ACTA1,PPP3CA
BPA_1500µg	6.31E-04 to 1.85E-02	20.% (35/168)	BLNK,APOE,GEM,MYCL1,BDNF,PTHLH,HRAS,FIP1L1,CD74,CDK9,LDHB,PTK2,COL1A2,PRPS1,EGR2,AKT1,BCKDHA,CFB,ACTA1,PPP3CA,GRIN1,NONO,USP15,COL2A1,SLC34A2,RNF17,DEDD2,ROCK1,HSPA8,RND3,GAB1,CD68,UQCRC2,ALDOA,COL9A2
BPA_4500µg	2.6E-05 to 3.13E-02	5.6% (7/125)	ROCK1,PTK2,GEM,RND3,NRCAM,HRAS,SPRED2
**Cardiovascular Disease**			
BPA_500µg	5.89E-04 to 2.97E-02	6.9% (7/101)	BDNF,PIK3CG,SYK,RHOA,FGF8,SERPIND1,SP4
BPA_1500µg	1.31E-02 to 1.85E-02	4.2% (7/168)	APOE,GRIN1,AKT1,BDNF,PIK3CG,CFB,SP4
BPA_4500µg	7.68E-04 to 3.13E-02	7.2% (9/125)	APOE,IGF2,MYH6,APOB,PPARD,CFB,CYSLTR1,SERPIND1,SP4
**Reproductive System Disease**			
BPA_500µg	4.56E-03 to 3.6E-02	6.9% (7/101)	CD2AP,SDCBP,DDEF1,MAD2L1BP,SYK,RHOA,FGF8
BPA_1500µg	2.42E-03 to 2.09E-02	7.7% (13/168)	DENND2D,AXIN1,PTHLH,FGF8,HRAS,SLIT2,TCF3,LRPAP1,FGF13,PTK2,AKT1,SYK,SERPINA1
BPA_4500µg	2.92E-04 to 3.13E-02	16% (20/125)	DENND2D,APOE,KRT7,MXRA8,TUBA4A,MEOX1,HRAS,RPA1,SLIT2,CD74,PROM1,PTK2,IGF2,BHLHB2,CALB2,CFB,SORT1,CYR61,DPH1,ITGB5
**Gastrointestinal Disease**			
BPA_500µg	1E-02 to 3.43E-02	9.9% (10/101)	RUNX3,EBP,SLC27A2,CLDN4,NDRG1,FGF8,CCNB2,DNAJB9,ACOT7,SET
BPA_1500µg	1.99E-03 to1.85E-02	14.9% (25/168)	APOE,SLC27A2,SRPX,FGF8,CCNB2,HRAS,KIAA0746,SET,COL1A2,AQP3,YES1,AKT1,CLDN4,PIK3CG,EFNB1,ALAS1,SERTAD2,ANKRD9,TFDP1,NDRG1,RRAD,COL2A1,SLIT2,DNAJB9,HSPA8

BPA_4500µg	1.58E-02 to 2.5E-02	8.0% (10/125)	YWHAG,NDRG1,KRT7,AXIN1,TUBA4A,HRAS,CD74,SLIT2,CYR61,ACTG1
**Genetic Disorder**			
BPA_500µg	1E-02 to 2.97E-02	9.9% (10/101)	EBP,TJP2,PRPS1,EGR2,PARK7,KCNJ1,CYB5R1,MRE11A,ACTA1,HMBS
BPA_1500µg	1.99E-03 to 1.91E-02	8.3% (14/168)	APOE,NSDHL,MAGT1,KRT17,PTHLH,COL2A1,SETX,PFKM,EFNB1,SOX3,ERCC5,JARID1C,ACTA1,NPC2
BPA_4500µg	1.58E-02 to 3.13E-02	11.2% (14/125)	APOE,APOB,PKD2,CYB5R1,EXT1,HRAS,SETX,FLCN,EGR2,PARK7,EFNB1,GFAP,CTH,TBXAS1
**Skeletal and Muscular Disorders**			
BPA_500µg	1E-02 to 2.97E-02	4.0% (4/101)	RUNX3,EBP,BDNF,ACTA1
BPA_1500µg	1.99E-03 to 1.85E-02	13.7% (23/168)	BLNK,APOE,GRIN1,NONO,USP15,BDNF,PTHLH,COL2A1,FIP1L1,CD74,CDK9,DEDD2,LDHB,HSPA8,EGR2,PRPS1,CD68,UQCRC2,CFB,ALDOA,COL9A2,PPP3CA,ACTA1
BPA_4500µg	1.27E-02 to 3.13E-02	5.6% (7/125)	IGF2,PPP1R13L,AXIN1,BHLHB2,TTC5,CFLAR,TBXAS1
**Hematological Disease**			
BPA_500µg	1E-02 to 2.97E-02	5.9% (6/101)	BLNK,BDNF,RHOA,PIK3CG,SYK,CYB5R1
BPA_1500µg	6.48E-03 to 2.05E-02	8.9% (15/168)	BLNK,E2F4,MLLT10,BDNF,CREBBP,NUP214,CD74,CDK9,TCF3,YES1,CDK8,AKT1,PIK3CG,SYK,PPP3CA
BPA_4500µg	4.94E-03 to 3.13E-02	4.8% (6/125)	BLNK,APOE,APOB,CYB5R1,TCF3,SPRED2
