# Supplementary material for: Toxicogenomic and Phenotypic Analyses of Bisphenol-A Early-Life Exposure Toxicity in Zebrafish
Source: PLoS One. 2011 Dec 14;6(12):e28273. doi: 10.1371/journal.pone.0028273 (PMC3237442; doi:10.1371/journal.pone.0028273)
Supplement: Table S5 — Selected top functional subcategories of ‘Canonical Pathways’ that are significantly enriched with human homologs of zebrafish genes deregulated by 500 µg/L, 1500 µg/L and 4500 µg/L of BPA. (PDF) [file pone.0028273.s007.pdf]

Table S5. Selected top functional subcategories of 'Canonical Pathways' that are significantly enriched with human homologs of zebrafish genes deregulated by 500 µg/L, 1500 µg/L and 4500 µg/L of BPA. The data was generated using Ingenuity Pathway Analysis™ software and only subcategories with *P* <0.05 (Fisher's Exact Test) and having at least 4% of homologs enriched in two treatment groups are listed.

Functional Subcategories	Percentage (no. molecules enriched / no. molecules used in analysis)	Molecules (human homologs of zebrafish genes) used in the analysis
**Tight Junction Signaling**		
BPA_500µg	7.9% (8/101)	MYLK, TJP2, CLDN4, RHOA, MYL4, MYH11, ACTG1, ACTA1
BPA_1500µg	3% (5/168)	AKT1, CLDN4, PPP2R2A, ACTG1, ACTA1
BPA_4500µg	4.8% (6/125)	MYH6, JAM3, TGFB2, MYL4, MYH11, ACTG1
**Actin Cytoskeleton Signaling**		
BPA_500µg	8.9% (9/101)	MYLK, TIAM2, PIK3CG, RHOA, FGF8, MYL4, MYH11, ACTG1, ACTA1
BPA_1500µg	5.4% (9/168)	PTK2, ROCK1, TIAM2, PIK3CG, FGF8, HRAS, ACTG1, ACTA1, FGF13
BPA_4500µg	7.2% (9/125)	PTK2, ROCK1, MYH6, DIAPH3, MYL4, HRAS, MYH11, PIK3R2, ACTG1
**Integrin Signaling**		
BPA_500µg	7.9% (8/101)	MYLK, TSPAN3, RND3, ASAP1, PIK3CG, RHOA, ACTG1, ACTA1
BPA_1500µg	5.4% (9/168)	PTK2, ROCK1, AKT1, RND3, PIK3CG, TSPAN2, HRAS, ACTG1, ACTA1
BPA_4500µg	7.2% (9/125)	PTK2, ROCK1, TSPAN3, RND3, HRAS, CAPN2, PIK3R2, ACTG1, ITGB5
**B Cell Receptor Signaling**		
BPA_500µg	5.9% (6/101)	BLNK, CAMK2D, GAB1, PIK3CG, SYK, PPP3CA
BPA_1500µg	4.8% (8/168)	BLNK, AKT1, CAMK2D, GAB1, PIK3CG, SYK, HRAS, PPP3CA
BPA_4500µg	4.8% (6/125)	BLNK, CAMK2D, HRAS, PIK3R2, PPP3CA, CALM1
**NRF2-mediated Oxidative Stress Response**		
BPA_500µg	5% (5/101)	PIK3CG, DNAJB9, ACTG1, ACTA1, DNAJC11
BPA_1500µg	6% (10/168)	AKT1, PIK3CG, CREBBP, DNAJC3, HRAS, DNAJC10, DNAJB9, ACTG1, DNAJB5, ACTA1
BPA_4500µg	4.8% (6/125)	DNAJC18, HRAS, DNAJC10, PIK3R2, ACTG1, DNAJB5
**Ephrin Receptor Signaling**		
BPA_500µg	4% (4/101)	SDCBP, AXIN1, PIK3CG, RHOA
BPA_1500µg	5.4% (9/168)	PTK2, ROCK1, GRIN1, GNB4, AKT1, AXIN1, PIK3CG, EFNB1, HRAS

BPA_4500µg	6.4% (8/125)	PTK2, ROCK1, GNB4, SDCBP, AXIN1, EFNB1, HRAS, EPHA3
**Axonal Guidance Signaling**		
BPA_500µg	6.9% (7/101)	WNT8A, SDCBP, BDNF, PIK3CG, RHOA, MYL4, PPP3CA
BPA_1500µg	6.5% (11/168)	PTK2, ROCK1, GNB4, AKT1, BDNF, PIK3CG, EFNB1, RTN4, HRAS, SLIT2, PPP3CA
BPA_4500µg	10.4% (13/125)	HRAS, SLIT2, EPHA3, PTK2, ROCK1, GNB4, MICAL1, SDCBP, EFNB1, RTN4, MYL4, PIK3R2, PPP3CA
**VEGF Signaling**		
BPA_500µg	3% (3/101)	PIK3CG, ACTG1, ACTA1
BPA_1500µg	4.2% (7/168)	PTK2, ROCK1, AKT1, PIK3CG, HRAS, ACTG1, ACTA1
BPA_4500µg	4% (5/125)	PTK2, ROCK1, HRAS, PIK3R2, ACTG1
**Clathrin-mediated Endocytosis**		
BPA_500µg	5.9% (6/101)	CD2AP, PIK3CG, FGF8, ACTG1, ACTA1, PPP3CA
BPA_1500µg	4.2% (7/168)	HSPA8, PIK3CG, FGF8, ACTG1, ACTA1, PPP3CA, FGF13
BPA_4500µg	3.2% (4/125)	PIK3R2, ACTG1, ITGB5, PPP3CA
**Synaptic Long Term Potentiation**		
BPA_500µg	2% (2/101)	CAMK2D, PPP3CA
BPA_1500µg	4% (6/168)	GRIN1, CAMK2D, PPP1R3C, CREBBP, HRAS, PPP3CA
BPA_4500µg	4% (5/125)	CAMK2D, PPP1R3C, HRAS, PPP3CA, CALM1
**Glycolysis/Gluconeogenesis**		
BPA_500µg	3% (3/101)	ADH6, HK1, PGM1
BPA_1500µg	4% (6/168)	ADH6, C10ORF134, ENO3, ALDOA, LDHB, PFKM
BPA_4500µg	4% (5/125)	ADH6, C10ORF134, ENO3, PGM1, LDHB
