# Supplementary material for: Toxicogenomic and Phenotypic Analyses of Bisphenol-A Early-Life Exposure Toxicity in Zebrafish
Source: PLoS One. 2011 Dec 14;6(12):e28273. doi: 10.1371/journal.pone.0028273 (PMC3237442; doi:10.1371/journal.pone.0028273)
Supplement: Table S6 — Human homolog of zebrafish genes that were significantly deregulated in all three BPA exposed groups (500 µg/L, 1500 µg/L and 4500 µg/L). (PDF) [file pone.0028273.s008.pdf]

Table S6. Human homolog of zebrafish genes (14) that were significantly deregulated in all three BPA exposed groups (500 µg/L, 1500 µg/L and 4500 µg/L) and has been reported to be endocrine-regulated. References below table.

Gene Symbol [Description]	Function	Endocrine-regulated
ZNF384 [zinc finger protein 384; CIZ/Nmp4]	transcription regulator	Parathyroid (Alvarez et al. 2005; Shah et al. 2004)
EGR2 [early growth response 2; Krox20]	transcription regulator	E2 (Pedram et al. 2007)
SP4 [specificity protein 4]	transcription regulator	E2 (Pedram et al. 2007)
GABPA [GA binding protein transcription factor, alpha subunit; NRF2a]	transcription regulator	Thyroid (Rodríguez-Peña et al. 2002) and E2 (Rodríguez-Cuenca et al. 2007)
RND3 [Rho family GTPase 3]	enzyme (GTPase)	E2 (Bektic et al. 2004)
BLNK [B-cell linker]	signal transducer	E2 (Frasor et al., 2004)
HSD17B8 [hydroxysteroid (17-beta) dehydrogenase 8]	enzyme (metabolic)	E2 and E2 metabolism (Rotinen et al. 2009)
LRRC4 [leucine rich repeat containing 4]	others	Antithyroid (Propylthiouracil) (Dong et al. 2007)
CPA2 [carboxypeptidase A2 (pancreatic)]	enzyme (peptidase)	E2 (Pedram et al. 2007)
SLC34A2 [solute carrier family 34, member 2; NAPI2]	transporter	E2 (Xu et al. 2003) and Thyroid (Alcalde et al. 1999)
NDRG1 [N-myc downstream regulated 1]	enzyme (kinase)	E2 (Frasor et al., 2004)
SSR2 [signal sequence receptor, beta]	others	E2 (Ivanga et al. 2007)
ACADM [acyl-Coenzyme A dehydrogenase; MCAD]	enzyme (metabolic)	E2 (Ivanga et al. 2007)

Reference

Alcalde AI, Sarasa M, Raldúa D, Aramayona J, Morales R, Biber J,et al. 1999. Role of thyroid hormone in regulation of renal phosphate transport in young and aged rats. Endocrinology 140:1544-1551.

Alvarez M, Shah R, Rhodes SJ, Bidwell JP.2005. Two promoters control the mouse Nmp4/CIZ transcription factor gene. Gene. 347:43-54.

Bektic J, Wrulich OA, Dobler G, Kofler K, Ueberall F, Culig Z, et al. 2004. Identification of genes involved in estrogenic action in the human prostate using microarray analysis. Genomics 83:34-44.

Dong H, Yauk CL, Williams A, Lee A, Douglas GR, Wade MG. 2007. Hepatic gene expression changes in hypothyroid juvenile mice: characterization of a novel negative thyroid-responsive element. Endocrinology148:3932-3940.

Frasor J, Stossi F, Danes JM, Komm B, Lyttle CR, Katzenellenbogen BS. 2004. Selective estrogen receptor modulators: discrimination of agonistic versus antagonistic activities by gene expression profiling in breast cancer cells. Cancer Res 64:1522-1533.

Ivanga M, Labrie Y, Calvo E, Belleau P, Martel C, Luu-The V, et al. 2007. Temporal analysis of E2 transcriptional induction of PTP and MKP and downregulation of IGF-I pathway key components in the mouse uterus. Physiol Genomics 29:13-23.

Pedram A, Razandi M, Aitkenhead M, Hughes CC, Levin ER. 2002. Integration of the non-genomic and genomic actions of estrogen: membrane-initiated signaling by steroid to transcription and cell biology. J Biol Chem 277:50768-50775.

Rodríguez-Peña A, Escrivá H, Handler AC, Vallejo CG. 2002. Thyroid hormone increases transcription of GA-binding protein/nuclear respiratory factor-2 alpha-subunit in rat liver. FEBS Lett 514:309-314.

Rodríguez-Cuenca S, Monjo M, Gianotti M, Proenza AM, Roca P. 2007. Expression of mitochondrial biogenesis-signaling factors in brown adipocytes is influenced specifically by 17beta-estradiol, testosterone, and progesterone. Am J Physiol Endocrinol Metab 292:E340-346.

Rotinen M, Celay J, Alonso MM, Arrazola A, Encio I, Villar J.2009.Estradiol induces type 8 17beta-hydroxysteroid dehydrogenase expression: crosstalk between estrogen receptor alpha and C/EBPbeta. J Endocrinol 200:85-92.

Shah R, Alvarez M, Jones DR, Torrungruang K, Watt AJ, Selvamurugan N,et al. 2004. Nmp4/CIZ regulation of matrix metalloproteinase 13 (MMP-13) response to parathyroid hormone in osteoblasts. Am J Physiol Endocrinol Metab 287:E289-96.

Xu H, Uno JK, Inouye M, Xu L, Drees JB, Collins JF, et al. 2003.Regulation of intestinal NaPi-IIb cotransporter gene expression by estrogen. Am J Physiol Gastrointest Liver Physiol 285:G1317-1324.
