# Supplementary material for: Toxicogenomic and Phenotypic Analyses of Bisphenol-A Early-Life Exposure Toxicity in Zebrafish
Source: PLoS One. 2011 Dec 14;6(12):e28273. doi: 10.1371/journal.pone.0028273 (PMC3237442; doi:10.1371/journal.pone.0028273)
Supplement: Table S7 — Selected functional subcategories that are significantly (Fisher's Exact Test P<0.05) enriched with human homologs of zebrafish genes deregulated in all BPA treatment groups (500 µg/L, 1500 µg/L and 4500 µg/L). (PDF) [file pone.0028273.s009.pdf]

Table S7. Selected functional subcategories that are significantly (Fisher's Exact Test *P* <0.05) enriched with human homologs of zebrafish genes deregulated in all BPA treatment groups (500 µg/L, 1500 µg/L and 4500 µg/L).The data was generated using Ingenuity Pathway Analysis™ software and only subcategories enriched with known endocrine-regulated homologs are listed. Functional annotation are referenced below the table.

Functional Subcategories	Functional Annotation (reference)	Molecules (human homologs)
**Cell Morphology**	remodeling of dendrites (Ramos et al. 2007)	SP4
	branching of fibroblast cell lines (Katoh et al. 2002)	RND3
	morphology of neuromuscular junctions (O' leary et al. 2007)	GABPA
	permeability of mitochondrial membrane (Unoki and Nakamura, 2003)	EGR2
	size of B lymphocytes (Pappu et al. 1999)	BLNK
**Cell-To-Cell Signaling and Interaction**	neurotransmission of neuromuscular junctions (O' leary et al. 2007)	GABPA
	dynamics of focal adhesions (Klein et al. 2008)	RND3
**Cellular Assembly and Organization**	remodeling of dendrites, quantity of dendrites (Ramos et al. 2007)	SP4
	permeability of mitochondrial membrane (Unoki and Nakamura, 2003)	EGR2
**Cellular Development**	development of trigeminal motor neurons;differentiation of Schwann cells;myelination of neurons (Schneider-Maunoury et al. 1997; Herdegen and Leah, 1998; Le et al. 2005)	EGR2
	growth of gonadal cell lines (Unoki and Nakamura, 2001)	EGR2
	development of mononuclear leukocytes (Minegishi et al. 1999; Lefebvre et al. 2005)	BLNK, EGR2
	development of thymocytes (Lefebvre et al. 2005)	EGR2
	differentiation of B lymphocytes (Flemming et al. 2003)	BLNK
**Gene Expression**	activation of gene (Sawada et al. 1999; Herdegen and Leah, 1998; Torrungruang et al. 2002)	EGR2, GABPA, ZNF384
	expression of endoplasmic reticulum stress element (Abdelrahim et al. 2005)	SP4
**Cell Death**	survival of trophoblast cells (Chen et al. 2006)	NDRG1
	apoptosis (Boswell et al. 2007;Jacobs et al. 2007; Stein et al 2004; Tan et al. 2001; Zorick et al. 1999)	BLNK, EGR2, GABPA, NDRG1, RND3,
**Cellular Growth and Proliferation**	growth of colorectal cancer cell lines;growth of gonadal and endometrial cell lines (Unoki and Nakamura, 2001)	EGR2
	proliferation of Schwann cells (Zorick et al. 1999)	EGR2

	proliferation of pre-B lymphocytes (Flemming et al. 2003)	BLNK
	colony formation of prostate cancer cell lines (Bandyopadhyay et al. 2003)	NDRG1
	proliferation of glioma cells (Wu et al. 2008)	LRRC4
**Tissue Morphology**	fusion of cranial nerve ganglion (Herdegen and Leah, 1998)	EGR2
	degeneration of sciatic nerve (Okuda et al. 2004)	NDRG1
	quantity of B-1a lymphocytes (Pappu et al. 1999)	BLNK
	quantity of Purkinje cells (Nguyên-Trân et al. 2000)	SP4
**Cellular Movement**	mingling of cells (Voiculescu et al. 2001)	EGR2
	guidance of motor axons (Helmbacher et al. 1998)	EGR2
**Lipid Metabolism**	metabolism of fatty acid (Fomitcheva et al. 1998; Matsubara et al. 1990)	HSD17B8, ACADM
**Molecular Transport**	nuclear export of molecule (Nakamoto et al. 2000)	ZNF384
	transport of phosphoric acid (Field et al. 1999)	SLC34A2
**Embryonic Development**	development of rhombomere 3, 5 and 6 (Schneider-Maunoury et al. 1997)	EGR2
	differentiation and survival of trophoblast cells (Chen et al. 2006)	NDRG1
**Nervous System Development and Function**	development of trigeminal motor neurons; myelination of neurons; fusion of cranial nerve ganglion; neurological process of peripheral nerve; proliferation and differentiation of Schwann cells (Schneider-Maunoury et al. 1997; Herdegen and Leah, 1998; Zorick et al. 1999: Le et al. 2005)	EGR2
	guidance of motor axons (Helmbacher et al. 1998; Zhang et al. 2005)	EGR2, LRRC4
	morphology and neurotransmission of neuromuscular junctions (O' leary et al. 2007)	GABPA
	remodeling of dendrites; quantity of dendrites; quantity of Purkinje cells (Ramos et al. 2007)	SP4
**Reproductive System Development and Function**	polyploidy of mammary cells (Kim et al. 2004)	NDRG1
	growth of gonadal cell lines (Unoki and Nakamura, 2001)	EGR2
	gonad development (Aziz et al. 2001)	HSD17B8
	spermatogenesis (Nakamoto et al. 2004)	ZNF384 homolog
**Endocrine System Development and Function**	metabolism of androgen and estrogen (Fomitcheva et al. 1998)	HSD17B8

**Skeletal and Muscular System Development and Function**	morphology and neurotransmission of neuromuscular junctions (O' leary et al. 2007)	GABPA
	suppressed the formation of mineralized nodules in osteoblastic cell (Shen et al. 2002)	ZNF384
**Tissue Development**	development of motor nucleus of trigeminal nerve; development of rhombomere 3, 5, and 6; myelination of peripheral nerve (Schneider-Maunoury et al. 1997; Herdegen and Leah, 1998)	EGR2
	differentiation of trophoblast (Chen et al. 2006)	NDRG1
**Hematological System Development and Function**	development of mononuclear leukocytes (Minegishi et al. 1999; Lefebvre et al. 2005)	BLNK, EGR2
	proliferation and differentiation of pre-B lymphocytes; quantity of B-1a lymphocytes; size of B lymphocytes (Flemming et al. 2003; Pappu et al. 1999)	BLNK
	arrest in development of thymocytes (Lefebvre et al. 2005)	EGR2
**Immune and Lymphatic System Development and Function**	development of lymphocytes (Minegishi et al. 1999; Lefebvre et al. 2005)	BLNK, EGR2
	proliferation and differentiation of pre-B lymphocytes; quantity of B-1a lymphocytes; size of B lymphocytes (Flemming et al. 2003; Pappu et al. 1999)	BLNK
	arrest in development of thymocytes (Lefebvre et al. 2005)	EGR2
**Cancer**	developmental process of colorectal cancer cell lines (Guan et al. 2000: Unoki and Nakamura, 2001)	EGR2, NDRG1
	neoplasia and metastasis of liver; polyploidy of bladder cell lines; differentiation and polyploidy of colorectal cancer cell lines; colony formation of prostate cancer cell lines (Bandyopadhyay et al. 2003; Guan et al. 2000; Kim et al. 2004)	NDRG1
	growth of endometrial and colorectal cancer cell lines (Unoki and Nakamura, 2001)	EGR2
	B-cell leukemia of mice (Hayashi et al. 2003)	BLNK
	suppress tumorigenesis and invasion of glioma cells (Wu et al. 2008)	LRRC4
**Neurological Disease**	apoptosis of Schwann cells; misrouting of axons; congenital hypomyelination; Dejerine-Sottas disease; Charcot-Marie-Tooth disease; polyneuropathy (Herdegen and Leah, 1998; Szigeti et al. 2008; Zorick et al. 1999)	EGR2
	neurological disorder of sciatic nerve (degeneration and demyelination of sciatic nerve) (Okuda et al. 2004)	NDRG1
	Alzheimer disease (Boutillier et al. 2007)	SP4

	suppress tumorigenesis, proliferation and invasion of glioma cells; hypermethylated in in brain tumors (Wu et al. 2008)	LRRC4
**Genetic Disorder**	congenital hypomyelination; Dejerine-Sottas disease; Charcot-Marie-Tooth disease (Szigeti et al. 2008)	EGR2
**Hematological Disease**	pre-B cell leukemia of mice (Hayashi et al. 2003)	BLNK
**Gastrointestinal Disease**	developmental process of colorectal cancer cell lines (Guan et al. 2000: Unoki and Nakamura, 2001)	EGR2, NDRG1
	growth of colorectal cancer cell lines (Unoki and Nakamura, 2001)	EGR2
	neoplasia and metastasis of liver; differentiation and polyploidy of colorectal cancer cell lines (Guan et al. 2000; Kim et al. 2004)	NDRG1
**Cardiovascular Disease**	sinus bradycardia and ventricular tachycardia of mice (Nguyên-Trân et al. 2000)	SP4
**Connective Tissue Disorders**	connective tissue disorder (Corut et al. 2006; Jang et al. 2006; Sela et al. 2008)	BLNK, EGR2, SLC34A2
**Reproductive System Disease**	colony formation of prostate cancer cell lines; polyploidy of mammary cells (Bandyopadhyay et al. 2003; Kim et al. 2004)	NDRG1

Reference

Abdelrahim M, Liu S, Safe S. Induction of Endoplasmic Reticulum-induced Stress Genes in Panc-1 Pancreatic Cancer Cells Is Dependent on Sp Proteins. J Biol Chem 2005 Apr 22;280(16):16508-13.

Aziz N, Anderson E, Lee GY, Woo DD. Arrested testis development in the cpk mouse may be the result of abnormal steroid metabolism. Mol Cell Endocrinol. 2001 Jan 22;171(1-2):83-8.

Bandyopadhyay S, Pai SK, Gross SC, Hirota S, Hosobe S, Miura K, Saito K, Commes T, Hayashi S, Watabe M, Watabe K. The drg-1 gene suppresses tumor metastasis in prostate cancer. Cancer Res 2003 Apr 15;63(8):1731-6.

Boswell SA, Ongusaha PP, Nghiem P, Lee SW. The Protective Role of a Small GTPase RhoE against UVB-induced DNA Damage in Keratinocytes. J Biol Chem 2007 Feb 16;282(7):4850-8.

Boutillier S, Lannes B, Buée L, Delacourte A, Rouaux C, Mohr M, Bellocq JP, Sellal F, Larmet Y, Boutillier AL, Loeffler JP. Sp3 and sp4 transcription factor levels are increased in brains of patients with Alzheimer's disease. Neurodegener Dis. 2007;4(6):413-23.

Chen B, Nelson DM, Sadovsky Y. N-Myc Down-regulated Gene 1 Modulates the Response of Term Human Trophoblasts to Hypoxic Injury. J Biol Chem 2006 Feb 03;281(5):2764-72.

Corut A, Senyigit A, Ugur SA, Altin S, Ozcelik U, Calisir H, Yildirim Z, Gocmen A, Tolun A. Mutations in SLC34A2 cause pulmonary alveolar microlithiasis and are possibly associated with testicular microlithiasis. Am J Hum Genet. 2006 Oct;79(4):650-6.

Feild JA, Zhang L, Brun KA, Brooks DP, Edwards RM. Cloning and functional characterization of a sodium-dependent phosphate transporter expressed in human lung and small intestine. Biochem Biophys Res Commun 1999 05 19;258(3):578-82.

Flemming A, Brummer T, Reth M, Jumaa H. The adaptor protein SLP-65 acts as a tumor suppressor that limits pre-B cell expansion. Nat Immunol 2003 01 1;4(1):38-43.

Fomitcheva J, Baker ME, Anderson E, Lee GY, Aziz N. Characterization of Ke 6, a new 17beta-hydroxysteroid dehydrogenase, and its expression in gonadal tissues. J Biol Chem 1998 08 28;273(35):22664-71

Guan RJ, Ford HL, Fu Y, Li Y, Shaw LM, Pardee AB. Drg-1 as a differentiation-related, putative metastatic suppressor gene in human colon cancer. Cancer Res 2000 Feb 1;60(3):749-55.

Hayashi K, Yamamoto M, Nojima T, Goitsuka R, Kitamura D. Distinct Signaling Requirements for D&mgr; Selection, IgH Allelic Exclusion, Pre-B Cell Transition, and Tumor Suppression in B Cell Progenitors. Immunity 2003 Jun;18(6):825-836.

Helmbacher F, Pujades C, Desmarquet C, Frain M, Rijli FM, Chambon P, Charnay P. Hoxa1 and Krox-20 synergize to control the development of rhombomere 3. Development 1998 Dec 1;125(23):4739-48.

Herdegen T, Leah JD. Inducible and constitutive transcription factors in the mammalian nervous system: control of gene expression by Jun, Fos and Krox, and CREB/ATF proteins. Brain Res Brain Res Rev 1998 Dec;28(3):370-490.

Jacobs AT, Marnett LJ. Heat Shock Factor 1 Attenuates 4-Hydroxynonenal-mediated Apoptosis: critical role for heat shock protein 70 induction and stabilization of Bcl-XL. J Biol Chem 2007 Nov 16;282(46):33412-20.

Jang J, Lim DS, Choi YE, Jeong Y, Yoo SA, Kim WU, Bae YS. MLN51 and GM-CSF involvement in the proliferation of fibroblast like synoviocytes in the pathogenesis of rheumatoid arthritis. Arthritis Res Ther 2006 Jan 01;8(6):R170.

Katoh H, Harada A, Mori K, Negishi M. Socius is a novel Rnd GTPase-interacting protein involved in disassembly of actin stress fibers. Mol Cell Biol 2002 May 1;22(9):2952-64.

Kim KT, Ongusaha PP, Hong YK, Kurdistani SK, Nakamura M, Lu KP, Lee SW. Function of Drg1/Rit42 in p53-dependent Mitotic Spindle Checkpoint. J Biol Chem 2004 Sep 10;279(37):38597-602.

Klein RM, Spofford LS, Abel EV, Ortiz A, Aplin AE. B-RAF Regulation of Rnd3 Participates in Actin Cytoskeletal and Focal Adhesion Organization. Mol Biol Cell 2008 Feb 01;19(2):498-508.

Le N, Nagarajan R, Wang JY, Svaren J, LaPash C, Araki T, Schmidt RE, Milbrandt J. Nab proteins are essential for peripheral nervous system myelination. Nat Neurosci 2005 07 1;8(7):932-40.

Lefebvre JM, Haks MC, Carleton MO, Rhodes M, Sinnathamby G, Simon MC, Eisenlohr LC, Garrett-Sinha LA, Wiest DL. Enforced expression of Spi-B reverses T lineage commitment and blocks beta-selection. J Immunol 2005 May 15;174(10):6184-94.

Matsubara Y, Narisawa K, Miyabayashi S, Tada K, Coates PM, Bachmann C, Elsas LJ, Pollitt RJ, Rhead WJ, Roe CR. Identification of a common mutation in patients with medium-chain acyl-CoA dehydrogenase deficiency. Biochem Biophys Res Commun 1990 08 31;171(1):498-505.

Minegishi Y, Rohrer J, Coustan-Smith E, Lederman HM, Pappu R, Campana D, Chan AC, Conley ME. An essential role for BLNK in human B cell development. Science 1999 Dec 3;286(5446):1954-7.

Nakamoto T, Yamagata T, Sakai R, Ogawa S, Honda H, Ueno H, Hirano N, Yazaki Y, Hirai H. CIZ, a zinc finger protein that interacts with p130(cas) and activates the expression of matrix metalloproteinases. Mol Cell Biol 2000 Mar 1;20(5):1649-58.

Nakamoto T, Shiratsuchi A, Oda H, Inoue K, Matsumura T, Ichikawa M, Saito T, Seo S, Maki K, Asai T, Suzuki T, Hangaishi A, Yamagata T, Aizawa S, Noda M, Nakanishi Y, Hirai H. Impaired spermatogenesis and male fertility defects in CIZ/Nmp4-disrupted mice. Genes Cells. 2004 Jun;9(6):575-89.

Nguyên-Trân VT, Kubalak SW, Minamisawa S, Fiset C, Wollert KC, Brown AB, Ruiz-Lozano P, Barrere-Lemaire S, Kondo R, Norman LW, Gourdie RG, Rahme MM, Feld GK, Clark RB, Giles WR, Chien KR. A novel genetic pathway for sudden cardiac death via defects in the transition between ventricular and conduction system cell lineages. Cell 2000 Sep 1;102(5):671-82.

Okuda T, Higashi Y, Kokame K, Tanaka C, Kondoh H, Miyata T. Ndrg1-deficient mice exhibit a progressive demyelinating disorder of peripheral nerves. Mol Cell Biol 2004 May 01;24(9):3949-56.

O'leary DA, Noakes PG, Lavidis NA, Kola I, Hertzog PJ, Ristevski S. Targeting of the ETS Factor Gabp{alpha} Disrupts Neuromuscular Junction Synaptic Function. Mol Cell Biol 2007 May 01;27(9):3470-80.

Pappu R, Cheng AM, Li B, Gong Q, Chiu C, Griffin N, White M, Sleckman BP, Chan AC. Requirement for B cell linker protein (BLNK) in B cell development. Science 1999 Dec 3;286(5446):1949-54.

Ramos B, Gaudillière B, Bonni A, Gill G. Transcription factor Sp4 regulates dendritic patterning during cerebellar maturation. Proc Natl Acad Sci U S A 2007 Jun 05;104(23):9882-7.

Sawada J, Simizu N, Suzuki F, Sawa C, Goto M, Hasegawa M, Imai T, Watanabe H, Handa H. Synergistic transcriptional activation by hGABP and select members of the activation transcription factor/cAMP response element-binding protein family. J Biol Chem 1999 Dec 10;274(50):35475-82.

Schneider-Maunoury S, Seitanidou T, Charnay P, Lumsden A. Segmental and neuronal architecture of the hindbrain of Krox-20 mouse mutants. Development 1997 Mar 1;124(6):1215-26.

Sela U, Dayan M, Hershkoviz R, Lider O, Mozes E. A Peptide that ameliorates lupus up-regulates the diminished expression of early growth response factors 2 and 3. J Immunol 2008 Feb 01;180(3):1584-91.

Shen ZJ, Nakamoto T, Tsuji K, Nifuji A, Miyazono K, Komori T, Hirai H, Noda M. Negative regulation of bone morphogenetic protein/Smad signaling by Cas-interacting zinc finger protein in osteoblasts. J Biol Chem 2002 Aug 16;277(33):29840-6.

Stein S, Thomas EK, Herzog B, Westfall MD, Rocheleau JV, Jackson RS, Wang M, Liang P. NDRG1 Is Necessary for p53-dependent Apoptosis. J Biol Chem 2004 Nov 19;279(47):48930-40.

Szigeti K, Wiszniewski W, Saifi GM, Sherman DL, Sule N, Adesina AM, Mancias P, Papasozomenos SCh, Miller G, Keppen L, Daentl D, Brophy PJ, Lupski JR. Functional, histopathologic and natural history study of neuropathy associated with EGR2 mutations. Neurogenetics. 2007 Nov;8(4):257-62.

Tan JE, Wong SC, Gan SK, Xu S, Lam KP. The adaptor protein BLNK is required for b cell antigen receptor-induced activation of nuclear factor-kappa B and cell cycle entry and survival of B lymphocytes. J Biol Chem 2001 Jun 8;276(23):20055-63.

Torrungruang K, Alvarez M, Shah R, Onyia JE, Rhodes SJ, Bidwell JP. DNA binding and gene activation properties of the Nmp4 nuclear matrix transcription factors. J Biol Chem 2002 May 3;277(18):16153-9.

Unoki M, Nakamura Y. EGR2 induces apoptosis in various cancer cell lines by direct transactivation of BNIP3L and BAK. Oncogene 2003 Apr 10;22(14):2172-85.

Unoki M, Nakamura Y. Growth-suppressive effects of BPOZ and EGR2, two genes involved in the PTEN signaling pathway. Oncogene 2001 Jul 27;20(33):4457-65.

Voiculescu O, Taillebourg E, Pujades C, Kress C, Buart S, Charnay P, Schneider-Maunoury S. Hindbrain patterning: Krox20 couples segmentation and specification of regional identity. Development 2001 Dec 1;128(24):4967-78.

Wu M, Chen Q, Li D, Li X, Li X, Huang C, et al.. LRRC4 inhibits human glioblastoma cells proliferation, invasion, and proMMP-2 activation by reducing SDF-1 alpha/CXCR4-mediated ERK1/2 and Akt signaling pathways. J Cell Biochem. 2008. 103:245-255.

Zhang Q, Wang J, Fan S, Wang L, Cao L, Tang K, Peng C, Li Z, Li W, Gan K, Liu Z, Li X, Shen S, Li G. Expression and functiona characterization of LRRC4, a novel brain-specific member of the LRR superfamily. FEBS Lett. 2005 Jul 4;579(17):3674-82.

Zorick TS, Syroid DE, Brown A, Gridley T, Lemke G. Krox-20 controls SCIP expression, cell cycle exit and susceptibility to apoptosis in developing myelinating Schwann cells. Development 1999 Apr 1;126(7):1397-406.
